# Identifying differentiation markers between dermal fibroblasts and adipose-derived mesenchymal stromal cells (AD-MSCs) in human visceral and subcutaneous tissues using single-cell transcriptomics

**DOI:** 10.1186/s13287-025-04185-w

**Published:** 2025-02-11

**Authors:** Magdalena Koczkowska, Anna Kostecka, Małgorzata Zawrzykraj, Kamil Myszczyński, Aneta Skoniecka, Milena Deptuła, Agata Tymińska, Katarzyna Czerwiec, Marcin Jąkalski, Jacek Zieliński, David K. Crossman, Michael R. Crowley, Mirosława Cichorek, Piotr M. Skowron, Michał Pikuła, Arkadiusz Piotrowski

**Affiliations:** 1 3P-Medicine Laboratory, Medical University of Gdansk, Gdansk, Poland; 2 Department of Biology and Pharmaceutical Botany, Medical University of Gdansk, Gdansk, Poland; 3 Division of Clinical Anatomy, Department of Anatomy, Medical University of Gdansk, Gdansk, Poland; 4 Centre of Biostatistics and Bioinformatics Analysis, Medical University of Gdansk, Gdansk, Poland; 5 Division of Embryology, Department of Anatomy, Medical University of Gdansk, Gdansk, Poland; 6 Department of Surgical Oncology, Transplant Surgery and General Surgery, Medical University of Gdansk, Gdansk, Poland; 7 Genomic Core Facility, University of Alabama at Birmingham, Birmingham, AL USA; 8 Department of Molecular Biotechnology, Faculty of Chemistry, University of Gdansk, Gdansk, Poland

**Keywords:** Single-cell RNA sequencing, scRNA-seq, Human adipose-derived mesenchymal stromal cells, AD-MSCs, Dermal fibroblasts, Visceral AD-MSCs, Subcutaneous AD-MSCs, Regenerative medicine, Heterogeneity

## Abstract

**Background:**

Adipose-derived mesenchymal stromal cells (AD-MSCs) and fibroblasts are both widely used in regenerative medicine, demonstrating significant potential for personalized cell therapy. A major challenge in their use lies in their high biological similarity, encompassing morphology, differentiation capabilities, and flow cytometric markers, making their distinction difficult.

**Methods:**

In our study, we aimed to compare AD-MSCs obtained from two types of adipose tissue, subcutaneous and visceral, alongside skin fibroblasts. Notably, all tissue samples were sourced from the same donors. We analyzed the cells for surface antigens via flow cytometry and conducted single-cell RNA sequencing, followed by verification with quantitative PCR (qPCR).

**Results:**

Our results revealed phenotypic similarities between the isolated AD-MSCs and dermal fibroblasts, particularly in the expression of markers characteristic of AD-MSCs. However, through in-depth analyses, we identified distinct differences between these cell types. Specifically, we pinpointed 30 genes exhibiting the most significant variations in expression between AD-MSCs and fibroblasts. These genes are associated with biological processes such as tissue remodeling, cell movement, and activation in response to external stimuli. Among them, *MMP1*,* MMP3*,* S100A4*,* CXCL1*,* PI16*,* IGFBP5*,* COMP* were further validated using qPCR, clearly demonstrating their potential to differentiate between AD-MSCs and fibroblasts.

**Conclusions:**

Our scRNA-seq analysis elucidates the transcriptional landscape of AD-MSCs and fibroblasts with unprecedented resolution, highlighting both the population-specific markers and the intrapopulation heterogeneity. Our findings underscore the importance of employing high-resolution techniques for cell identification.

**Supplementary Information:**

The online version contains supplementary material available at 10.1186/s13287-025-04185-w.

## Graphical Abstract

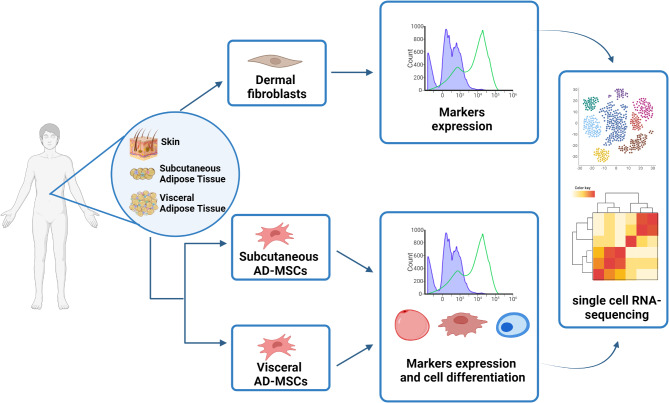

Created with BioRender.co.

## Background

Adipose-derived mesenchymal stromal cells (AD-MSCs) have attracted significant attention in both pre-clinical and clinical research due to their potential in treating a wide array of conditions, ranging from various autoimmune diseases such as inflammatory bowel disease, systemic sclerosis, type 1 diabetes, and rheumatoid arthritis, to neurological disorders including amyotrophic lateral sclerosis, ischemic stroke, and spinal cord injuries. Additionally, their role in facilitating tissue repair processes, such as in osteoarthritis, wound healing, and vascular restoration, has been extensively studied [1, 2]. The biological characteristics of AD-MSCs may vary depending on numerous factors, including the anatomical location of the cells and the donor’s health status [3].

To address the variability and ensure consistency in research and clinical application, the International Federation for Adipose Therapeutics and Science (IFATS) and the International Society for Cellular Therapy (ISCT) have introduced guidelines for the standardized identification of cultured AD-MSCs [2, 4–6]. These guidelines recommend evaluating surface markers and the cells’ ability to differentiate into adipocytes, osteocytes, and chondrocytes. Despite these efforts, the variability introduced by different cell processing steps, such as isolation, culture, and storage, poses challenges for the reproducibility and translation of research findings to clinical applications [7].

The heterogeneity within AD-MSC populations not only raises safety concerns for their therapeutic use but also prompts questions about the characteristics of the intended cell product. Although AD-MSCs show promising therapeutic potential, the mechanisms behind their effects are not yet fully understood [8]. The introduction of single-cell RNA sequencing (scRNA-seq) have enabled the exploration of cellular diversity within tissues, allowing for the identification of distinct cell subpopulations and advancing our understanding of adipose tissue spatial organization and heterogeneity [9]. High-resolution transcriptomics approaches have been applied to characterize signatures associated with metabolic diseases and embolic risk of AD-MSCs infusion, their differentiation capacity and wound healing properties [10–14].

Fibroblasts play a crucial role in the formation of connective tissue in the human body. Their primary function is to produce and maintain the extracellular matrix (ECM) by secreting essential components such as collagen, elastin, fibronectin, proteoglycans and glycosaminoglycans [15]. During tissue regeneration, fibroblasts migrate to injury sites, facilitate ECM production, and regulate inflammation through cytokine and growth factor release [16]. Importantly, fibroblasts share certain surface markers with AD-MSCs, complicating the selection of target cell types for therapeutic applications if there is cross-contamination in fibroblast or AD-MSCs cell cultures [17]. Moreover, fibroblasts can be induced to differentiate into adipocytes, osteocytes, and chondrocytes, making them a subject of interest in regenerative medicine and tissue engineering [17, 18]. Due to their properties, fibroblasts have been extensively examined in regenerative medicine for wound and ulcer healing, tissue engineering for tissue and organ repair, as well as the treatment of immune disorders [19–21]. Given the similarities between AD-MSCs and fibroblasts, distinguishing between these cell types using reliable markers and high-resolution transcriptomic profiling is essential for assessing their clinical utility.

Here, we performed a single-cell expression profiling study of human adipose tissue and dermal fibroblasts from 11 samples collected from four unrelated adult individuals. This included AD-MSCs from two distinct anatomical locations, i.e. visceral and subcutaneous adipose tissues, as well as the corresponding dermal fibroblasts. This approach has provided a detailed catalog of cell types present in adipose tissue and elucidated transcriptomic differences between AD-MSCs and fibroblasts, offering valuable insights into their respective roles and potential in clinical applications.

## Methods

### Sample collection

Fragments of human subcutaneous (SAT) and visceral (VAT) adipose tissues, and skin samples were collected from four adult oncological patients during scheduled surgical procedures. Only individuals with body mass index (BMI) ≤ 30, without diabetes and other autoimmune disorders, were included in the study. Furthermore, these individuals had not undergone neoadjuvant chemotherapy or radiotherapy for at least five years before sample collection.

### Isolation and culture of AD-MSCs

The procedure for isolating AD-MSCs was performed as previously described [22]. In brief, after enzymatic isolation and lysis of erythrocytes, the cells were suspended in Dulbecco’s modified Eagle medium (DMEM low glucose-1000 mg/L) supplemented with 10% fetal bovine serum FBS (Sigma-Aldrich, USA) and 1% penicillin/streptomycin (Sigma Aldrich, USA). The obtained cells were then seeded into 75 cm^2^ flasks (Primaria Corning, USA), and cultured under 37° C and 5% CO_2_ conditions for 24 h. After this, the medium was changed to remove non-adherent cells. Subsequently, the medium was changed every 24 h following each passage, and every two days thereafter. When the cells reached 70–80% confluence, they were trypsinzed using 0.25% trypsin/EDTA solution (Sigma- Aldrich, USA). For each passage, 0.375 × 10^6^ cells were seeded. The AD-MSCs were cultured for a total of three weeks, reaching the third passage. After trypsinization at the third passage, the cells were centrifuged, counted, and cryopreserved in liquid nitrogen at a density of 1 × 10^6^ cells per sample, in preparation for transcriptomic analysis.

### Isolation and cell culture of dermal fibroblasts

Human skin samples were washed in PBS containing antibiotics (100 units/ ml penicillin and 100 µg/ml streptomycin) and then cut into small fragments. Fibroblasts were obtained from the skin explants culture through a culture process. Initially, the epidermis was removed by digesting the samples with dispase (6 units/ml), and the resulting human dermis was placed in 6-well plates containing DMEM (high glucose 4,5 g/L) medium supplemented with 10% FBS and penicillin/streptomycin (100 units/ ml penicillin and 100 µg/ml streptomycin). The medium was refreshed every 2–3 days, and the first passage of cells was conducted after three weeks of culture. At the third passage, 1 × 10^6^ actively proliferating cells were collected and cryopreserved for subsequent transcriptomic analysis.

### AD-MSCs and fibroblasts surface marker analysis by flow cytometry

Flow cytometry was applied to assess the presence of stromal/stem cell markers in AD-MSCs and fibroblasts, according to the IFATS and ISCT guidelines [2, 4–6]. The expression of positive markers *NT5E* (*CD73*), *THY1* (*CD90*), and *ENG* (*CD105*), as well as negative markers *CD14*, *CD19*, and *TPRC* (*CD45*), was evaluated. Additionally, the positive *ANPEP* (*CD13*) and negative marker *PECAM1* (*CD31*) were also checked. The presence of the same markers was verified in fibroblasts.

For surface marker detection, AD-MSCs and fibroblasts at the third passage were trypsinized, washed, and stained with monoclonal antibodies (1 × 10^4^ cells per assay). The following antibodies (all from Beckman Coulter, France) were used: NT5E (CD73) (clone AD-2/PE), THY1 (CD90) (clone Thy-1/310/APC750), ENG (CD105) (clone TEA3/17.1.1 /PC7), CD14 (clone RMO52 /ECD), CD19 (clone J3-119 /KO), CD31 (clone 5.6E/FITC), CD45 (clone J33/KO), CD13 (clone Immu103.44/ PC5.5) and DAPI (PB450). The cells were incubated with the respective antibodies for 30 minutes (min) at room temperature (RT). Following incubation, the cells were washed, resuspended in PBS, and analyzed using the CytoFLEX flow cytometer (Beckman Coulter, USA). Data analysis was performed with Kaluza software (Beckman Coulter, USA). The analyzed cells were separated from debris and gated as AD-MSC/ fibroblast, next single cells (singlets) were selected (on a FSC-A versus FSC-H) to exclude signaling data from doublets. Unstained cells were used to determine background autofluorescence.

### AD-MSCs chondrogenic, osteogenic and adipogenic differentiation

Chondrogenesis differentiation was assessed by micro pellet formation. AD-MSCs at the second passage were seeded in 96-well plates at a density of 8 × 10^4^ cells per well in 200 µl of differentiation medium (StemPro Chondrogenesis Differentiation Kit, Thermo Fisher Scientific, USA). The experiment included four biological replicates of subcutaneous adipose derived mesenchymal stromal cells (SASC) and three biological replicates of visceral adipose derived mesenchymal stromal cells (VASC). AD-MSCs seeded in MesenPro RS medium (Thermo Fisher Scientific, USA) represented the control cells. The medium was refreshed every two days, and the cells were differentiated for 14 days. Subsequently, the cells were washed with PBS and fixed in 4% paraformaldehyde (PFA, Santa Cruz Biotechnology, USA) for 30 min. To confirm differentiation, the cells were stained with 1% Alcian Blue pH 2.5 (Sigma-Aldrich, USA) for 30 min. Following staining, the cells were observed and photographed under an inverted microscope (Leica AG, Germany and Gryphax Software, Jenoptik AG, Germany). Alcian Blue staining was used to indicate the synthesis of proteoglycans by chondrocytes [23].

For osteogenic and adipogenic differentiation, AD-MSCs at the second passage were seeded in 96-well plates at a density of 5 × 10^3^ per well in 200 µl of differentiation medium (StemPro™ Osteogenesis Differentiation Kit, StemPro™ Adipogenesis Differentiation Kit, Thermo Fisher Scientific, USA). Similar to the chondrogenesis differentiation experiment, the experiment for osteogenic and adipogenic differentiation also included four biological replicates of SASC and three of VASC. AD-MSCs cultured in MesenPro RS medium (Thermo Fisher Scientific, USA) were used as a control. The medium was replaced every second day for 14 days. Subsequently, the cells were washed in PBS and fixed in 4% PFA. Differentiated cells were stained with Alizarin Red (Sigma Aldrich, USA) to visualize calcium deposits produced by osteoblast-like cells and Red Oil (Sigma Aldrich, USA) to detect lipid droplets [22]. Following staining, the cells were observed and photographed.

### Single-cell RNA library preparation and sequencing

SASC, VASC and fibroblasts samples frozen at the third passage were thawed in a 37°C water bath, washed with DMEM supplemented with 10% FBS (Sigma Aldrich, USA) and centrifuged at 300 x g for 5 min. The pellets were washed and then resuspended in PBS with 0.04% BSA. The viability and concentration of the cells were assessed using the EVE automatic cell counter (Nano EnTek, South Korea). The samples were adjusted by adding PBS with 0.04% BSA to achieve the final concentration of 1-1.5 × 10^6^ cells per milliliter. All samples tested negative for mycoplasma contamination. The capturing and library construction were performed using the Chromium Next GEM Single Cell 3’ v.3.1 library preparation kit (10x Genomics, USA), according to the manufacturer’s protocol. The single-cell libraries were sequenced on Illumina NextSeq 550 platform (Illumina, USA) in a 2 × 150 bp paired-end mode.

### scRNA-seq data processing

Sequencing results were demultiplexed and converted to FASTQ format using Illumina bcl2fastq software. Raw gene expression data in the form of Unique Molecular Identifiers (UMI) counts matrices were generated using the standard Cell Ranges software (v5.01) from 10xGenomics with the GRCh38 version of the human reference genome used for mapping. Further data processing was performed using the Seurat R package (4.1.3 version) unless otherwise specified [24]. This included initial filtering of low-quality cells and/or potential doublets, as well as the removal of apoptotic cells (cells undergoing cell death due to stress related to prior laboratory procedures). Specifically, cells with less than 2000 overall reads, cells expressing less than 1000 uniquely expressed genes, as well as cells with more than 10% mitochondrial RNA were excluded from further analyses. After quality control steps, each read count matrix was normalized to the total expression, multiplied by a scaling factor of 10,000, and log-transformed. Clustering analysis was performed using the first 40 principal components (PCs). To identify and remove doublets, the DoubletFinder package was applied [25].

Following the removal of doublets and quality filtering, the single-cell expression data from all samples were merged using the top 5000 shared highly variable genes based on standardized variance values. The merged gene expression datasets were integrated using Harmony R package [26]. To minimize the impact of cell cycle differences on downstream analysis, the cells cycle heterogeneity was normalized by calculating cell cycle phase scores based on canonical markers and regressing the scores out [27]. Principal Component Analysis (PCA) was performed on the gene expression data, followed by batch correction to address any remaining batch effects. The Louvain algorithm was applied as a modularity optimization technique for iterative cell clustering, and then a Uniform Manifold Approximation and Projection (UMAP) was generated based on the first 40 PCs, allowing for the identification of distinct clusters or cell populations that were further used in differential gene expression analysis.

### Differential gene expression analyses

Cellular identity was determined by finding positive and negative known gene markers for each cluster. Differentially expressed genes (DEGs) for each cluster and sample type were identified using the FindMarkers function in Seurat [24]. The default Wilcoxon Rank Sum test was applied within each identified cell type to determine DEGs. The resulting DEGs were filtered using a minimum log fold change of 1.0 and a Bonferroni-adjusted p-value threshold of < 0.05, unless stated otherwise. Gene ontology enrichment analysis was performed based on the identified DEGs using the topGO package and enrichR [28] to help to determine the functional annotations and biological processes associated with DEGs. The analysis results from the single-cell sequencing data were visualized using Seurat [24], the ggplot [29] and ggpubr [30] packages.

### Quantitative PCR verification analysis of AD-MSCs and fibroblasts markers

Total RNA was transcribed into cDNA using the Transcriptor First Strand cDNA Synthesis Kit (Roche Diagnostics GmbH, Germany). Quantitative real-time PCR (qPCR) was then performed on the Light Cycler 480 using the Light Cycler 480 SYBR Green I Master Kit (Roche Diagnostics GmbH, Germany). Seven target genes (*MMP1*,* MMP3*,* S100A4*,* CXCL1*,* PI16*,* IGFBP5*,* COMP*) were evaluated in AD-MSCs and fibroblasts, with *HPRT1*, *RPLP0*, and *RPLP13A* used as reference genes. Based on the results, relative gene expression was calculated using the 2^−ΔΔCT^ method. Gene-specific primers are presented in the Supplementary Table S1.

### Code Availability

The code used to process the data described in this study is available online at: https://github.com/FunctionalGenes/ASC_SC.git.

## Results

### Phenotype and differentiation capacity of cultured SASC, VASC and dermal fibroblasts

**Fig. 1:**
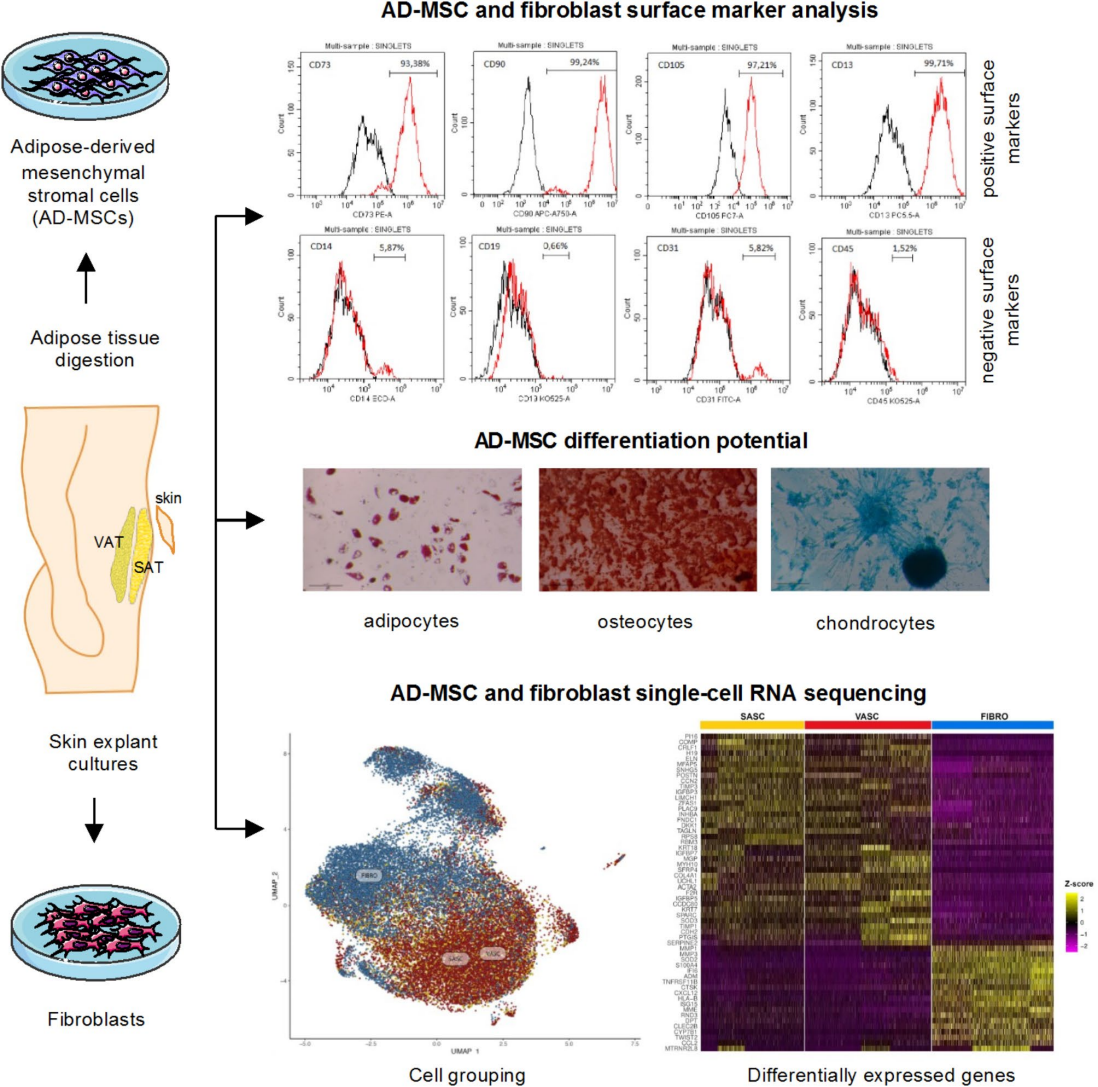
Overview of the experimental workflow Adipose-derived mesenchymal stromal cells (AD-MSCs) were harvested from subcutaneous adipose tissue (SAT) and visceral adipose tissue (VAT) obtained from four adult individuals. The isolation of AD-MSCs was achieved through enzymatic digestion of the adipose tissue samples. The phenotypic characterization of the isolated AD-MSCs was performed using flow cytometry to analyze surface marker expression. The multipotency of the AD-MSCs was validated by inducing their differentiation into adipocytes, osteocytes, and chondrocytes, employing standard tri-lineage differentiation assays. Concurrently, fibroblasts were cultured from skin explants up to the third passage. The transcriptomic landscapes of both AD-MSCs and fibroblasts were then elucidated through single-cell RNA sequencing (scRNA-seq), with subsequent analysis of cell grouping and identification of differentially expressed genes

**Fig. 2:**
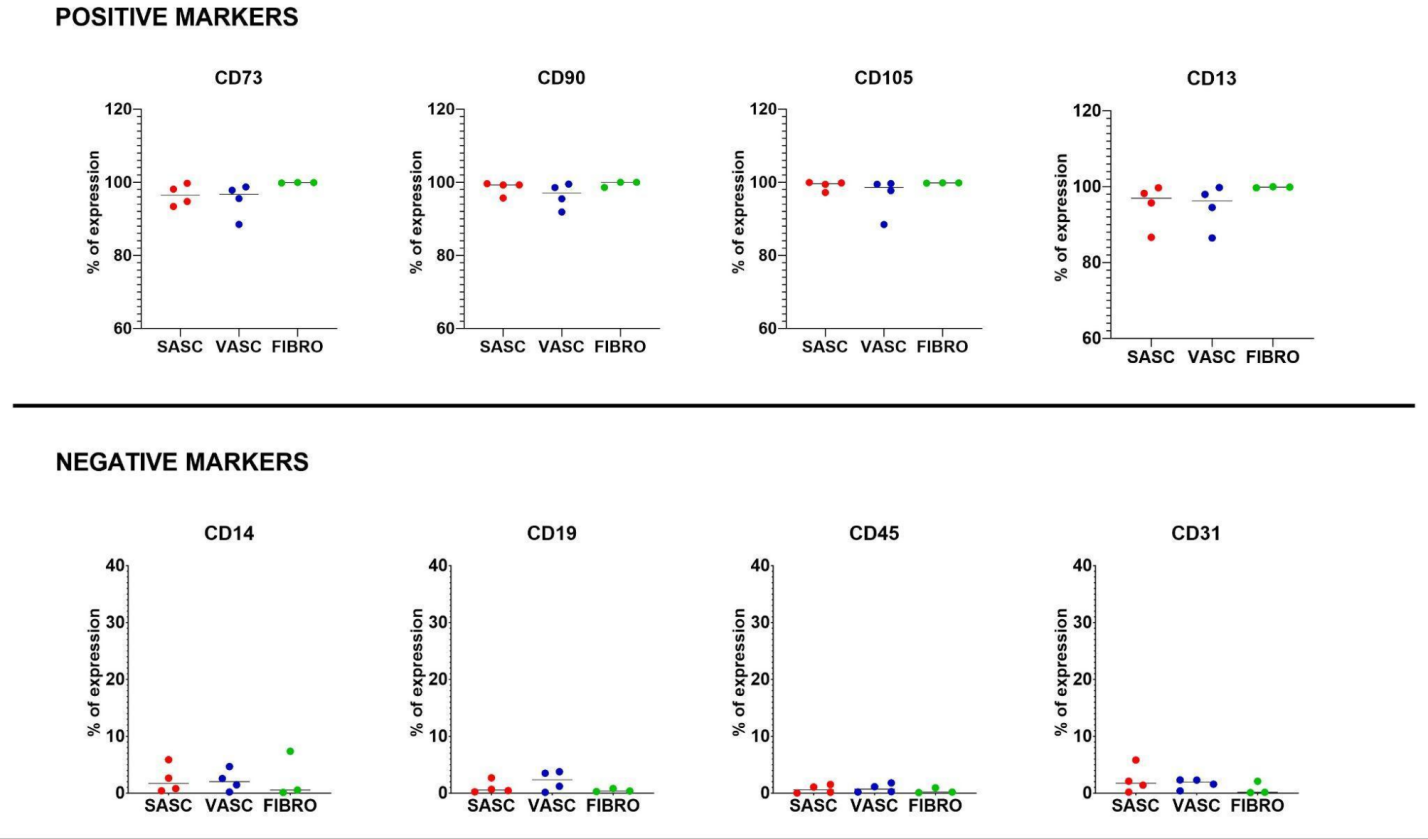
Expression of MSCs specific markers in SASC, VASC and FIBRO cell lines At passage 3, SASC, VASC and fibroblasts were evaluated for the expression of positive stem/stromal cell surface markers (CD73, CD90, CD105) and negative markers (CD14, CD19, CD45). Additional positive (CD13) and negative (CD31) markers were also assessed. Dots indicate samples obtained from four unrelated individuals for SASC and VASC, and from three unrelated individuals for FIBRO. The line represents the mean obtained for a specific marker

Immunophenotype profiling was performed on samples collected from four adult individuals enrolled in the study (Supp. Table S2). According to the minimal identification criteria established by ISCT, AD-MSCs were identified based on the expression of positive surface markers (CD73, CD90, CD105, CD13) and the low level of negative surface markers (CD14, CD45, CD19, CD31) [4]. Immunophenotype profiling of SASC and VASC confirmed the presence of a mesenchymal phenotype in cells derived from both adipose tissue locations across all individuals (Supp. Figure S1 and Supp. Table S3). Cell viability was assessed using DAPI staining, showing that at least 95% of the cells were viable (Supp. Table S3). Tri-lineage differentiation assays further confirmed the multi-lineage differentiation potential of studied VASC and SASC to differentiate into adipocytes, osteocytes, and chondrocytes (Supp. Figure S2). Additionally, fibroblast cells showed the presence of AD-MSCs stem/stromal markers, in line with the literature (Supp. Figure S3 and Supp. Table S3) [20, 31, 32]. The overview of the experimental workflow is presented in Fig. 1, while the phenotype analysis of AD-MSCs and fibroblasts is presented in Fig. 2.

### Single cell profiles of SASC and VASC

The total average number of viable cells was 2503 with a median of 5420 genes and 32,598 UMI counts per cell (Supp. Table S4 and Supp. Figure S4-S6). Expression-based clustering and visualization of the data in a two-dimensional space revealed nine distinct clusters within SASC and nine within VASC cells (Supp. Figure S7). The clusters showed variations in stromal/stem marker gene expression, with the highest percentage of positive cells (> 90% for at least three positive markers) detected in clusters 1–3, 5, 7 and 9 for both SASC and VASC (Supp. Figure S8). Overall, SASC and VASC were consistent with the results of flow cytometry, confirming the AD-MSCs phenotype.

We have identified a total of 281 genes that were differentially expressed between SASC and VASC (with an adjusted p-value < 0.05). Among these, only five genes - *SERPINE2* (serpin family E member 2) and *KRT18* (keratin 18), which were upregulated, and *PI16*,* CCN5*,* STMN2*, which were downregulated - demonstrated at least 1-fold difference in their geometrical mean expression (Supp. Table S5).

When analyzing clusters 1, 2, 3 and 8 of SASC, each cluster exhibited either slight upregulation or downregulation of genes associated with adipose progenitors (*CD36*,* PDGFRA*,* APOE*,* MGP* and *CEBPB*) or adipocyte differentiation (*CFD*) with at least a 0.15 log_2_ fold difference (Supp. Table S6). These findings are in agreement with independent studies [11, 13, 33]. Furthermore, we observed slightly elevated expression of osteoblast-related genes, such as *GPNMB*, *RUNX1* and *MMP14*, primarily in cluster 2 of SASC (Supp. Table S6). Gene Ontology (GO) terms pointed to muscle contraction, lipid biosynthesis, regulation of wound healing and RNA processing as predominant processes in these clusters (Supp. Table S7).

Cells within VASC clusters 2, 5, 7 and 9 showed higher expression of progenitor marker genes (*C1S*, *EIF1*, *DPP4*, *PDGFRA1*, *ADH1B* and *ICAM1)*, whereas cluster 4 was associated with a downregulation of *CFD*, a marker of adipocyte differentiation (Supp. Table S8) [13]. GO analysis associated these clusters with cell differentiation, RNA processing and extracellular matrix organization processes (Supp. Table S9).

### Single cell profiles of dermal fibroblasts

In parallel with AD-MSCs analysis, we performed scRNA-seq on matched dermal fibroblasts from three unrelated individuals (Supp. Table S2). After confirming the fibroblast phenotype within all seven clusters identified through expression-based clustering and visualizing the data in a two-dimensional space (Supp. Figure S7 and Supp. Figure S9), we also assessed the levels of stromal/stem marker gene expression in these clusters. The results were consistent with those obtained through flow cytometry (Supp. Figure S3 and Supp. Figure S10).

**Fig. 3:**
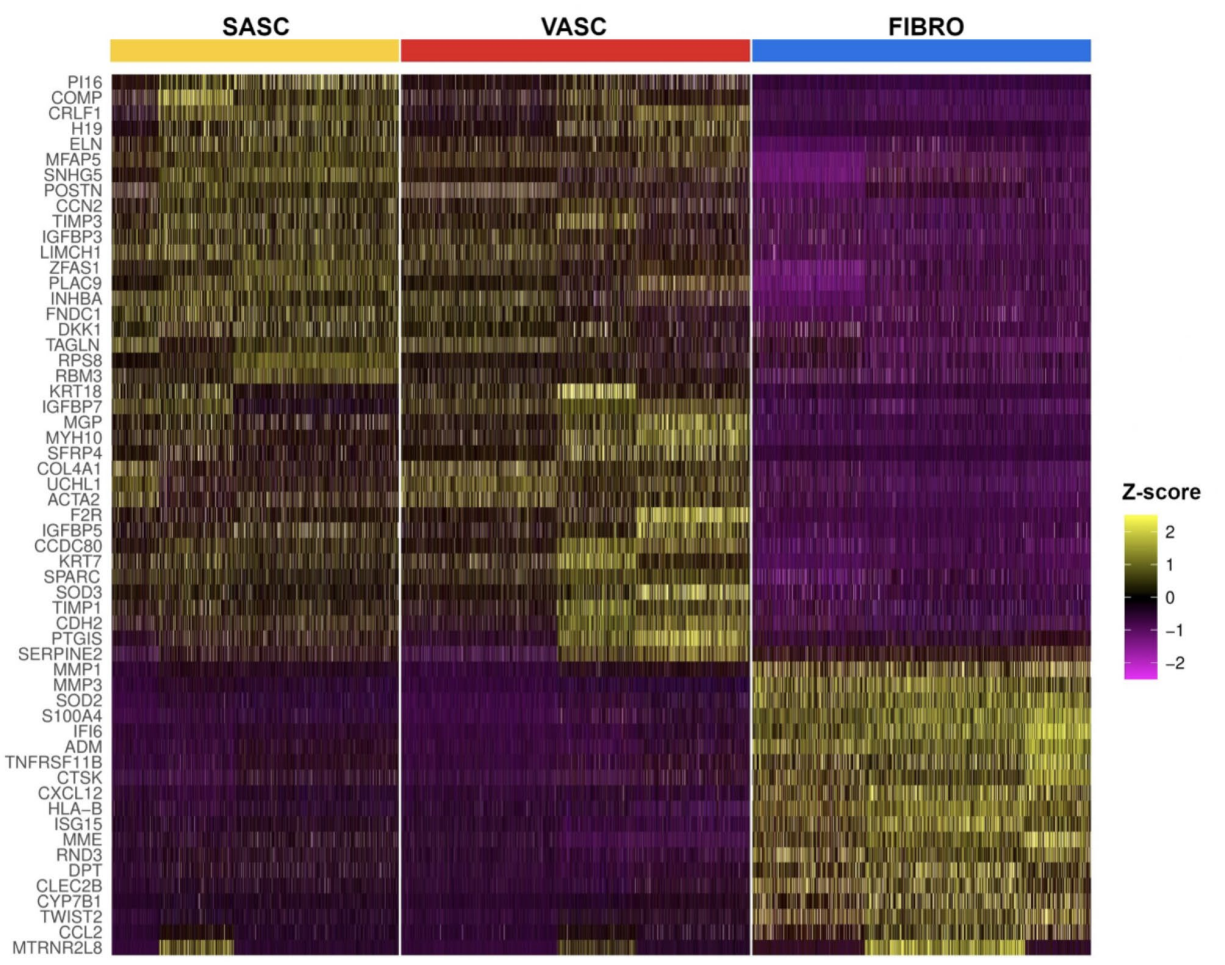
Heatmap illustrating the top differentially expressed genes (DEGs) across SASC, VASC and FIBRO Gene expression variability is quantified as z-scores, which denote the number of standard deviations from the mean expression level. The x-axis categorizes individual cells sampled from the SASC, VASC, and FIBRO groups. Columns represent single cells, while rows correspond to the respective genes. The color gradient from purple to yellow indicates expression levels from low (purple) to high (yellow)

We identified 1509 genes that are differentially expressed in fibroblasts when compared to SASC or VASC (an adjusted p-value < 0.05) (Supp. Table S10 and Fig. 3). We identified a refined subset of 10 upregulated and 20 downregulated fibroblast genes, all showing a log_2_ fold change of at least 1.5 (adjusted p-value < 0.001). These genes displayed consistent expression in both SASCs and VASCs and were in agreement with at least two independent studies with overlapping transcript sets and similar research focus (Supp. Table S11 and Supp. Table S12) [17, 34]. Notably, the upregulated genes in fibroblasts represent typical functions of this cell type and are associated with tissue remodeling and cell movement (*S100A4*,* MMP1*,* MMP3*,* CTSK*), activation of cells in response to external stimuli like infections or proinflammatory signals in general (*IFI6*,* ISG15*,* LY6E*,* CXCL1*) [35–41]. In contrast, the majority of downregulated genes in fibroblasts reflect cellular functions associated with AD-MSCs. The *COL4A1*,* SPARC*,* EFEMP1*,* CDH2*,* VCAN*,* TIMP3*,* POSTN*,* MGP*,* MFAP5* and *COMP* genes all play roles in extracellular matrix composition, cellular adhesion, and matrix remodeling [42–51]. They collectively influence cell-matrix interactions, tissue integrity, and differentiation potential of AD-MSCs. The *IGFBP7*,* IGFBP5* and *INHBA* genes are involved in growth factor signaling which influences cell proliferation, differentiation, and apoptosis [52–55]. Finally, the *TPM1*,* ACTA2* and *TAGLN* genes are involved in actin filament organization and influence cell morphology, motility, contraction, and potential differentiation pathways [56–58].

Among these 30 showing the most significant expression differences between AD-MSCs and dermal fibroblasts, *MMP1*,* MMP3*,* S100A4*,* CXCL1*,* PI16*,* IGFBP5*,* COMP* were further validated using qPCR, which clearly confirmed the direction of expression differences (Supp. Table S13).

## Discussion

Several notable studies have provided valuable insight into transcriptomic features of AD-MSCs and fibroblasts [17, 59, 60]. While these studies generally agreed on the high similarities between AD-MSCs and fibroblasts, attempts have been made to identify distinctive marker transcripts that could aid in differentiating between AD-MSCs and fibroblasts, as both cell types often co-exist within tissues [59, 61]. This distinction is an important issue to address because these two cell lineages are phenotypically very similar, yet they display different self-renewal and differentiation potential, which are of key importance in regenerative medicine research [18, 32, 62, 63].

Previous studies examining cell surface markers at the protein level have indicated that AD-MSCs and fibroblasts exhibit similar properties regarding surface antigens [61]. Lorenz et al. confirmed the presence of markers including CD73, CD90, CD105, CD29, CD44 by flow cytometry in both AD-MSCs and foreskin fibroblasts. Simultaneously, they assessed the absence of negative markers such as CD14, CD31, CD45, CD133 in both populations [64]. Additionally, in the study by Zanata et al., dermal fibroblasts displayed AD-MSCs-specific markers such as CD73, CD90, CD105 [18]. Similarly, our study demonstrated no statistically significant differences in the expression of positive (CD73, CD90, CD105, CD13) and negative markers (CD14, CD45, CD19, CD31) between AD-MSCs and dermal fibroblasts. These findings were further supported by transcriptomic analyses. Since both AD-MSCs and fibroblasts express MSCs markers, distinguishing between them using methods such as flow cytometry can be challenging.

However, at the molecular level, our study contributes novel insights to these attempts in two key aspects. Firstly, the use of scRNA-seq over bulk RNA-seq or narrow, targeted analyses such as quantitative polymerase chain reaction (qPCR) allowed for a higher resolution and sensitivity in detecting gene expression variation among individual cells. This inherent advantage of single-cell analysis unmasked the characteristics of distinct cell populations that escape detection by targeted or bulk experimental approaches. We were able to leverage quantitative differences in gene expression between different cell lineages. Consequently, in the absence of genes expressed exclusively in AD-MSCs or fibroblasts, we delineated a group of genes that were expressed in both fibroblasts and AD-MSCs, but with significantly varying expression levels, thus providing a solid foundation for differentiation between these two cell lineages. Secondly, our study design incorporated uniformly prepared and matched samples from four patients, encompassing both subcutaneous (SASC) and visceral (VASC) mesenchymal cells as well as their corresponding dermal fibroblasts from the same individuals. This standardized approach minimized the effects of environmental and genetic variations among donors and constituted the most representative sample set among similar studies to date.

AD-MSCs obtained from different sources may exhibit varying biological properties. The advanced age of the donor may reduce the immune properties of AD-MSCs [65]. Furthermore, obesity has been linked to a significant reduction in the biological activity of AD-MSCs, manifested through lower differentiation capacity and proangiogenic effect [66]. Additionally, stem cells obtained from patients with various diseases such as Parkinson’s disease, diabetes, and rheumatoid arthritis may function abnormally through impaired proliferation, secretion of pro-inflammatory cytokines, or increased senescence and apoptotic activity [67].

To further mitigate interindividual differences, we integrated findings from other studies for comparison with our results. This rigorous evaluation yielded a set of transcripts showing consistent expression patterns across studies, marking them as potential differentiation markers between fibroblasts and AD-MSCs (Supp. Table S12). Our research enabled the selection of 1509 genes whose expression significantly differed in AD-MSCs and fibroblasts. Among these, we identified genes that were upregulated and downregulated to the greatest extent in fibroblasts compared to SASC and VASC. Hence, these genes can serve as markers for the differential analysis of AD-MSCs and fibroblasts using other methods, such as qPCR or flow cytometry, what was also demonstrated in this study (Supp. Table S13).

Of particular significance are the differences in the expression of genes related to inflammation and the activity of metalloproteinases. Stem cells in our study exhibited significantly lower percentage of cells expressing *CXCL12* (32.7% in SASC and 20.8% in VASC, compared to 76.6% in fibroblasts), *CXCL1 (*0.8% in SASC and 1.4% in VASC, compared to 45.1% in fibroblasts*)*, as well as *MMP1 (*2.5% in SASC and 2.7% in VASC, compared to 83.7% in fibroblasts*)* and *MMP3* genes *(*13.4% in SASC and 5.5% in VASC, compared to 83.8% in fibroblast*).* Additionally, there was evident expression of the *COMP* gene (95.4% in SASC, 85.3% in VASC, compared to 22% in fibroblasts) and *ELN* (88.3% in SASC, 74.8% in VASC, compared to 36.5% in fibroblasts) compared to fibroblasts. This finding underscores the prominent role of fibroblasts in inflammatory processes and wound healing. The studied fibroblast population also exhibited the presence of other genes related to wound healing such as *CXCL8*, also known as *IL-8* (27.8% in fibroblasts, compared to 0.6% in SASC and 1.4% in VASC), *STC1* (45.7% in fibroblasts, compared to 3.7% in SASC and 3.3% in VASC), and *PTN* (65.7% in fibroblasts, compared to 14.7% and 15.5% in SASC and VASC, respectively). Nevertheless, the expression of *TIMP3* (98.1% in SASC and 95.7% in VASC, compared to 64.1% in fibroblasts), *MGP* (76.5% in SASC and 78.1% in VASC, compared to 5.4% in fibroblasts), *MFAP5* (98.9% in SASC and 97.3% in VASC, compared to 19.9% in fibroblasts) further suggests the potential involvement of AD-MSCs in processes related to the organization and production of the extracellular matrix, in line with a recently published human adipose tissue atlas [67]. In fact, clinical trials are currently underway to investigate the application of AD-MSCs in diseases characterized by excessive tissue fibrosis [68–70].

Currently, there are no sc-RNAseq studies in the existing literature that directly compare SASCs and VASCs cultured in vitro. Previous research has highlighted differences primarily at the tissue level, either immediately after isolation or across cells obtained from different sources. Our analyses confirm the heterogeneity within the SASC and VASC groups, demonstrating even subtle distinctions among SASC and VASC derived from the same donors. In both SASC and VASC cells populations, nine distinct clusters were detected, confirming the AD-MSCs heterogeneity. We identified 281 genes with differential expression between these groups, of which only five had statistically significant levels (genes that demonstrated at least 1-fold difference in their geometrical mean expression with an adjusted p-value < 0.05; Supp. Table S5). In contrast, Vijay et al. showed differences between cells isolated from SAT and VAT from donors with obesity [13]. Additionally, Wang et al. are focused on freshly isolated mesenchymal stem cells from adipose tissue, bone marrow, umbilical cord and foreskin, potentially containing other cells such as immune cells, endothelial cells, and fibroblasts [71].

Furthermore, the gene expression patterns observed in freshly isolated cells could still reflect the in vivo environment from which they were derived. Our research shows that in vitro cell culture provides a more standardized and uniform cell population. It is crucial to emphasize that AD-MSCs are predominantly expanded in vitro for clinical applications. Thus, our study is particularly relevant for evaluating the quality and biological activity of clinical-grade cell products. The insights from our research could be instrumental in ensuring the safety and efficacy of stem cell-based therapies, thereby offering substantial benefits to patients and fostering progress in the realm of regenerative medicine.

## Conclusions

Our scRNA-seq analysis elucidates the transcriptional landscape of AD-MSCs and fibroblasts with unprecedented resolution, highlighting both the population-specific markers and the intrapopulation heterogeneity. These findings could lead to more reliable lineage-tracing approaches and potentially enhance regenerative medicine strategies. Furthermore, our study enriches the ongoing debate regarding the differentiation and fate commitment of both MSCs and fibroblasts by emphasizing the necessity of high-resolution techniques like scRNA-seq for precise cell-type identification.

## Electronic supplementary material

Below is the link to the electronic supplementary material.

Supplementary Material 1

Supplementary Material 2

Supplementary Material 3

Supplementary Material 4

Supplementary Material 5

Supplementary Material 6

Supplementary Material 7

## Data Availability

Raw, de-identified RNA-sequencing files were deposited in the European Genome-Phenome Archive https://ega-archive.org/): accession no. EGAS00001006954.

## Abbreviations

AD-MSCs: Adipose-Derived Mesenchymal Stromal Cells
BMI: Body Mass Index
DEGs: Differentially Expressed Genes
ECM: Extracellular Matrix
FIBRO: Fibroblasts
GO: Gene Ontology
IFATS: The International Federation for Adipose Therapeutics and Science
ISCT: The International Society for Cellular Therapy
PCA: Principal Component Analysis
PCs: Principal Components
SASC: Subcutaneous Adipose-derived mesenchymal Stromal Cells
SAT: Subcutaneous Adipose Tissue
scRNA-seq: Single cell RNA sequencing
qPCR: Quantitative Polymerase Chain Reaction
UMI: Unique Molecular Identifiers
UMAP: Uniform Manifold Approximation and Projection
VASC: Visceral Adipose-derived mesenchymal Stromal Cells
VAT: Visceral Adipose Tissue
