# Supplementary material for: Identifying differentiation markers between dermal fibroblasts and adipose-derived mesenchymal stromal cells (AD-MSCs) in human visceral and subcutaneous tissues using single-cell transcriptomics
Source: Stem Cell Res Ther. 2025 Feb 11;16:64. doi: 10.1186/s13287-025-04185-w (PMC11818286; doi:10.1186/s13287-025-04185-w)
Supplement: Supplementary file 1 — Supplementary Material 1 [file 13287_2025_4185_MOESM1_ESM.docx]

**Supplementary Information**

**Identifying differentiation markers between dermal fibroblasts and adipose-derived mesenchymal stromal cells (AD-MSCs) in human visceral and subcutaneous tissues using single-cell transcriptomics**

Magdalena Koczkowska,^1,#^ Anna Kostecka,^1,2,#^ Małgorzata Zawrzykraj,^3,#^ Kamil Myszczyński,^4^ Aneta Skoniecka,^5^ Milena Deptuła,^5^ Agata Tymińska,^5^ Katarzyna Czerwiec,^3^ Marcin Jąkalski,^1^ Jacek Zieliński,^6^ David K. Crossman,^7^ Michael R. Crowley,^7^ Mirosława Cichorek,^5^ Piotr M. Skowron,^8^ Michał Pikuła,^5,*^ Arkadiusz Piotrowski,^1,2,*^

^1^ 3P-Medicine Laboratory, Medical University of Gdansk, Poland;

^2^ Department of Biology and Pharmaceutical Botany, Medical University of Gdansk, Poland;

^3^ Division of Clinical Anatomy, Medical University of Gdansk, Poland;

^4^ Centre of Biostatistics and Bioinformatics Analysis, Medical University of Gdansk, Poland;

^5^ Division of Embryology, Department of Anatomy, Medical University of Gdansk, Poland;

^6^ Clinic of Oncological Surgery, Medical University of Gdansk, Poland;

^7^ Genomic Core Facility, University of Alabama at Birmingham, Birmingham, Alabama, USA;

^8^ Department of Molecular Biotechnology, Faculty of Chemistry, University of Gdansk, Poland.

# These authors contributed equally: Magdalena Koczkowska, Anna Kostecka, Małgorzata Zawrzykraj

* These authors jointly supervised: Michał Pikuła, Arkadiusz Piotrowski

**Correspondence**

Arkadiusz Piotrowski, 3P-Medicine Laboratory, Medical University of Gdansk, 80-210 Gdansk, Poland. Email: [arkadiusz.piotrowski@gumed.edu.pl](mailto:arkadiusz.piotrowski@gumed.edu.pl)

Michał Pikuła, Division of Embryology, Department of Anatomy, Medical University of Gdansk, 80-210, Poland. E-mail: [michal.pikula@gumed.edu.pl](mailto:arkadiusz.piotrowski@gumed.edu.pl)

**Table of Contents:**

**Supplementary Tables**

**Note:** Supplementary Tables 5 - 10 are provided as separate Excel files due to their large size.

**Supp. Table S1.** Gene-specific primer sequences used for the qPCR verification analysis of AD-MSCs and fibroblasts markers.

**Supp. Table S2.** Summary of individuals characteristics and derived cell lines.

**Supp. Table S3**. Marker analysis by flow cytometry.

**Supp. Table S4.** Summary of quality control (QC) filtering steps within Seurat.

**Supp. Table S5**. List of differentially expressed genes (DEGs) between SASC and VASC.

**Supp. Table S6**. List of differentially expressed genes (DEGs) between clusters in SASC.

**Supp. Table S7**. Gene Ontology analysis in SASC clusters.

**Supp. Table S8**. List of differentially expressed genes (DEGs) between clusters in VASC.

**Supp. Table S9**. Gene Ontology analysis in VASC clusters.

**Supp. Table S10**. List of 1,509 differentially expressed genes (DEGs) between fibroblasts and SASC/VASC identified in this study.

**Supp. Table S11.** Detailed description of the top differentially expressed genes (DEGs) from SASC, VASC and dermal fibroblasts.

**Supp. Table S12.** List of the top 30 marker genes differentiating between fibroblasts and SASC/VASC.

**Supp. Table S13.** Results of confirmatory quantitative PCR analysis of selected AD-MSCs and fibroblast markers.

**Supplementary Figures**

**Supp. Figure S1.** Flow cytometry analysis of subcutaneous (SASC) and visceral (VASC) adipose-derived stromal cells.

**Supp. Figure S2.** Differentiation capacity of SASC and VASC collected from four unrelated individuals.

**Supp. Figure S3.** Flow cytometry analysis of dermal fibroblasts.

**Supp. Figure S4.** Quality control of single-cell RNA sequencing data of subcutaneous AD-MSCs collected from four individuals (SASC#1-SASC#4).

**Supp. Figure S5.** Quality control of single-cell RNA sequencing data of visceral AD-MSCs collected from four individuals (VASC#1-VASC#4).

**Supp. Figure S6.** Quality control of single-cell RNA sequencing data of dermal fibroblasts collected from three individuals (FIBRO#1-FIBRO#3).

**Supp. Figure S7.** Cell distribution and marker expression of SASC, VASC, and FIBRO.

**Supp. Figure S8.** Integrated expression of stem/stromal marker genes in clusters 1-9 of SASC and VASC from the four individuals analyzed in this study.

**Supp. Figure S9.** Integrated expression of positive and negative fibroblast marker genes in clusters 1-7 of dermal fibroblasts from the three individuals analyzed in this study.

**Supp. Figure S10.** Integrated expression of positive and negative stem/stromal marker genes in clusters 1-7 of dermal fibroblasts from the three individuals analyzed in this study.

**Supp. Table S1.** Gene-specific primer sequences used for the qPCR verification analysis of AD-MSCs and fibroblasts markers.

| **Gene** | **Forward primer (5’-3’)** | **Reverse primer (5’-3’)** |
| --- | --- | --- |
| *MMP 1* | GTTTTCTGGCCACAACTGCC | CCTTGGGGTATCCGTGTAGC |
| *MMP3* | GACCTGGAAATGTTTTGGCCC | AGCTTCAGTGTTGGCTGAGT |
| *S100A4* | CTCTCTCCTCAGCGCTTCTTC | TGTCCCTGTTGCTGTCCAAG |
| *CXCL1* | CTGGCGGATCCAAGCAAATG | GCCCCTTTGTTCTAAGCCAG |
| *PI16* | AGCGTGAGCACTACAACCTC | ACAGCCGATCCTCTCTGTCT |
| *IGFBP5* | CCCAATTGTGACCGCAAAGG | CGTCAACGTACTCCATGCCT |
| *COMP* | CCAAGTGGGCTACATCAGG | GTCCAAGACCACGTTGCTG |
| *RPL13A* | CCGAAGATGGCGGAGGTG | CGGAGGAAAGCCAGGTACTTC |
| *RPLP0* | CGTCCTCGTGGAAGTGACAT | CATGCGGATCTGCTGCATCT |
| *HPRT1* | CCTGGCGTCGTGATTAGTGAT | TCCAGCAGGTCAGCAAAGAA |

**Supp. Table S2.** Summary of individuals characteristics and derived cell lines.

| **ID** | **Sex ^a^** | **Age** | **BMI ^b^** | **Cancer type, TNM ^c^** | **Cell lines description ^d^** |
| --- | --- | --- | --- | --- | --- |
| #1 | M | 71 | 30 | Sigmoid colon cancer, cT2N0M1 | SASC#1 / VASC#1 / FIBRO#1 |
| #2 | F | 70 | 26 | Large intestine cancer, cT3N1M0 | SASC#2 / VASC#2 / FIBRO#2 |
| #3 | M | 67 | 22 | Stomach cancer, cT2N0M0 | SASC#3 / VASC#3 / FIBRO#3 |
| #4 | M | 60 | 29 | Stomach cancer, cT1N0M0 | SASC#4 / VASC#4 / - |

^a^ M - male, F - female; ^b^ Only individuals with body mass index (BMI) ≤30 were included in the study to avoid obesity-related bias; ^c^ Cancer TNM stage according to the current American Joint Committee on Cancer guidelines. ^d^ Subcutaneous and visceral adipose tissue, and skin samples were collected during scheduled surgical procedures, and processed to obtain subcutaneous adipose-derived mesenchymal stromal cells (SASC), visceral adipose-derived mesenchymal stromal cells (VASC) and fibroblasts (FIBRO).

**Supp. Table S3.** Marker analysis by flow cytometry.

Flow cytometry was applied to evaluate the expression of positive markers (CD73, CD90, CD105), negative markers (CD14, CD19, CD45), and additional markers (CD13, CD31) in subcutaneous adipose-derived mesenchymal stromal cells (SASC), visceral adipose-derived mesenchymal stromal cells (VASC), and dermal fibroblasts (FIBRO). The percentage of cells expressing the selected markers is presented in each column. Cell viability was assessed using DAPI (diamidino-2-phenylindole) staining. * The mean surface expression values per marker in SASC, VASC and FIBRO, along with the corresponding standard deviation (SD) values, are reported.

|  | Marker expression analysis (% of stained cells) | | | | | | | | |
| --- | --- | --- | --- | --- | --- | --- | --- | --- | --- |
|  | **CD73** | **CD90** | **CD105** | **CD14** | **CD19** | **CD45** | **CD13** | **CD31** | **DAPI** |
| SASC#1 | 93.38 | 99.24 | 97.21 | 5.87 | 0.66 | 1.52 | 99.71 | 5.82 | 2.30 |
| SASC#2 | 9812 | 95.69 | 99.43 | 0.80 | 0.44 | 0.03 | 86.66 | 1.41 | 2.24 |
| SASC#3 | 99.72 | 99.61 | 99.94 | 0.41 | 0.23 | 0.16 | 95.73 | 0.18 | 1.06 |
| SASC#4 | 94.74 | 99.28 | 99.81 | 2.64 | 2.69 | 1.07 | 98.22 | 2.1 | 1.80 |
| Mean*  ± SD | 96.49  2.90 | 98.46  1.90 | 99.10  1.30 | 2.43  2.50 | 1.01  1.10 | 0.70  0.70 | 95.08  5.80 | 2.38  2.40 | 1.85  0.60 |
| VASC#1 | 97.81 | 99.50 | 99.64 | 1.46 | 1.21 | 1.14 | 99.78 | 1.57 | 1.87 |
| VASC#2 | 95.56 | 91.87 | 97.70 | 2.57 | 3.77 | 0.20 | 94.52 | 2.30 | 3.93 |
| VASC#3 | 98.72 | 95.48 | 99.45 | 0.20 | 0.14 | 0.26 | 97.97 | 0.40 | 4.54 |
| VASC#4 | 88.47 | 98.56 | 88.44 | 4.67 | 3.51 | 1.80 | 86.48 | 2.32 | 1.16 |
| Mean*  ± SD | 95.14  4.60 | 96,.35  3.40 | 96.31  5.30 | 2.23  1.90 | 2.16  1.80 | 0.85  0.80 | 94.69  5.90 | 1.65  0.90 | 2.88  1.60 |
| FIBRO#1 | 99.90 | 99.98 | 99.84 | 0.12 | 0.28 | 0.18 | 99.72 | 0.10 | 0.44 |
| FIBRO#2 | 99.94 | 99.98 | 99.83 | 0.55 | 0.81 | 0.10 | 99.99 | 0.15 | 0.09 |
| FIBRO#3 | 99.80 | 98.59 | 99.75 | 7.36 | 0.37 | 0.94 | 99.88 | 2.09 | 1.45 |
| Mean*  ± SD | 99.88  0.07 | 99.52  0.80 | 99.81  0.05 | 2.68  4.06 | 0.49  0.28 | 0.41  0.46 | 99.86  0.14 | 0.78  1.13 | 0.66  0.71 |

**Supp. Table S4.** Summary of quality control (QC) filtering steps within Seurat.

**Individual #1**

|  | **SASC#1** | **VASC#1** | **FIBRO#1** |
| --- | --- | --- | --- |
| Number of cells before QC | 1875 (100%) | 6497 (100%) | 3711 (100%) |
| Number of cells with >10% of mitochondrial RNA | 271 (14.45%) | 944 (14.53%) | 438 (11.80%) |
| Number of cells with <1000 genes | 155 (8.27%) | 775 (11.93%) | 705 (18.99%) |
| Number of cells with <2000 reads | 100 (5.33%) | 632 (9.73%) | 691 (18.62%) |
| Final number of cells after QC | 1557 (83.04%) | 5323 (81.93%) | 2759 (74.35%) |
| Total genes detected | 24 290 (100%) | 25 221 (100%) | 23 434 (100%) |
| Number of genes expressed in >2 cells | 22 026 (90.68%) | 23 083 (91.52%) | 21 117 (90.11%) |

**Individual #2**

|  | **SASC#2** | **VASC#2** | **FIBRO#2** |
| --- | --- | --- | --- |
| Number of cells before QC | 4072 (100%) | 4369 (100%) | 5279 (100%) |
| Number of cells with >10% of mitochondrial RNA | 1144 (28.09%) | 1197 (27.40%) | 1919 (36.35%) |
| Number of cells with <1000 genes | 721 (17.71%) | 932 (21.33%) | 646 (12.24%) |
| Number of cells with <2000 reads | 664 (16.31%) | 863 (19.75%) | 526 (9.96%) |
| Final number of cells after QC | 2527 (62.06%) | 2667 (61.04%) | 3098 (58.69%) |
| Total genes detected | 24 361 (100%) | 24 973 (100%) | 24 432 (100%) |
| Number of genes expressed in >2 cells | 22 031 (90.44%) | 22 606 (90.52%) | 22 108 (90.49%) |

**Individual #3**

|  | **SASC#3** | **VASC#3** | **FIBRO#3** |
| --- | --- | --- | --- |
| Number of cells before QC | 3004 (100%) | 3935 (100%) | 2162 (100%) |
| Number of cells with >10% of mitochondrial RNA | 181 (6.03%) | 1002 (25.46%) | 285 (13.18%) |
| Number of cells with <1000 genes | 133 (4.43%) | 1659 (42.16%) | 312 (14.43%) |
| Number of cells with <2000 reads | 113 (3.76%) | 1574 (40.00%) | 285 (13.18%) |
| Final number of cells after QC | 2766 (92.08%) | 1897 (48.21%) | 1653 (76.46%) |
| Total genes detected | 24 838 (100%) | 23 963 (100%) | 23 255 (100%) |
| Number of genes expressed in >2 cells | 22 537 (90.74%) | 21 534 (89.86%) | 20 931 (90.01%) |

**Individual #4**

|  | **SASC#4** | **VASC#4** | **FIBRO#4** |
| --- | --- | --- | --- |
| Number of cells before QC | 2434 (100%) | 1801 (100%) | NA |
| Number of cells with >10% of mitochondrial RNA | 324 (13.31%) | 332 (18.43%) | NA |
| Number of cells with <1000 genes | 383 (15.74%) | 246 (13.66%) | NA |
| Number of cells with <2000 reads | 337 (13.85%) | 199 (11.05%) | NA |
| Final number of cells after QC | 1890 (77.65%) | 1400 (77.73%) | NA |
| Total genes detected | 24 793 (100%) | 25 261 (100%) | NA |
| Number of genes expressed in >2 cells | 22 552 (90.96%) | 22 970 (90.93%) | NA |

**Supp. Table S5.** List of differentially expressed genes (DEGs) between SASC and VASC.

**Note:** Supp. Table S5 is provided as a separate Excel file.

Five genes demonstrating at least 1-fold difference in their geometrical mean expression - *SERPINE2* (serpin family E member 2) and *KRT18* (keratin 18), which were upregulated, and *PI16, CCN5, STMN2*, which were downregulated - are presented in bold in column 1.

**Supp. Table S6.** List of differentially expressed genes (DEGs) between clusters in SASC.

**Note:** Supp. Table S6 is provided as a separate Excel file.

List of references cited in Suppl. Table S6 are provided in the Reference section in the Supplementary Information.

**Supp. Table S7.** Gene Ontology analysis in SASC clusters.

**Note:** Supp. Table S7 is provided as a separate Excel file.

**Supp. Table S8.** List of differentially expressed genes (DEGs) between clusters in VASC.

**Note:** Supp. Table S8 is provided as a separate Excel file.

Refence by Vijay et al. (2020) [PMID: 25068904] was cited in Supp. Table S8.

**Supp. Table S9.** Gene Ontology analysis in VASC clusters.

**Note:** Supp. Table S9 is provided as a separate Excel file.

**Supp. Table S10.** List of differentially expressed genes (DEGs) between fibroblasts and SASC/VASC identified in this study.

**Note:** Supp. Table S10 is provided as a separate Excel file.

**Supp. Table S11.** Detailed description of the top differentially expressed genes (DEGs) from SASC, VASC and dermal fibroblasts.

| **Gene (symbol)** | **Description** | **References** |
| --- | --- | --- |
| *Peptidase Inhibitor 16 (PI16)* | PI16 belongs to the catabolite activator protein (CAP). Associated with the extracellular matrix (ECM), inhibits peptidase activity and the growth of cells involved in myocardial development. It is also a type 2 metalloproteinase inhibitor under conditions of inflammation and high shear forces. | [7,8] |
| *Cartilage Oligomeric Matrix Protein (COMP)*  (MIM *600310) | Extracellular matrix protein, takes part in collagen folding and fibril formation. Involved in chondrogenesis, mechanical stress resistance, and thrombin inhibition. | [9,10] |
| *Cytokine Receptor-Like Factor 1 (CRLF1)*  (MIM *604237) | Participates in the ciliary neurotrophic factor receptor pathway. Associated with the inflammatory response. Its levels are increased by pro-inflammatory cytokines. Is expressed in the thymus, heart, bone marrow, and spleen. In addition, circular CRLF1 negatively regulates adipogenesis. | [11,12] |
| *Imprinted Maternally Expressed Noncoding Transcript (H19)*  (MIM *103280) | Long non-coding RNA, shows higher levels of expression in various cancers including breast, bladder, and oral squamous cell carcinoma. Associated with aging and inflammation. Participates in osteogenic differentiation. | [13,14] |
| *Elastin (ELN)*  (MIM *130160) | ELN proliferates mainly during prenatal development. In adulthood, its synthesis is inhibited. Elastin monomer-tropoelastin builds elastic fibers. Responsible for vascular smooth muscle proliferation and organization. It is found in the aorta, lungs, or ligaments. | [15,16] |
| *Microfibrillar-Associated Protein 5 (MFAP5)*  (MIM *601103) | MFAP5 is present in the microfibrils of the elastin crosslink in various tissues including adipose tissue. Is highly expressed in osteoblasts. Associated with cell signaling in microfibril assembling, elastogenesis, and cell viability. Furthermore, MFAP5 has pro-angiogenic effects. | [17,18] |
| *Small Nucleolar RNA Host Gene 5 (SNHG5)*  (MIM *613263) | Long non-coding RNA. Present in various disorders such as melanoma, gastric cancer, and colorectal cancer. Oncogene in the formation of liver cancers. | [19,20] |
| *Periostin (POSTN)*  (MIM *608777) | Associated with the extracellular matrix, it affects the differentiation and survival of osteoblasts. Found in bones, skin, and tendons. Predictive factor associated with poor prognosis in solid tumors. | [21–23] |
| *Cellular Communication Network Factor 2 (CCN2)*  (MIM *121009) | Present in endothelial cells, chondrocytes, fibroblasts or vascular smooth muscle cells. Participates in cell adhesion, proliferation and differentiation. Associated with the processes of angiogenesis, inflammation, osteogenesis and chondrogenesis. | [24–26] |
| *Tissue Inhibitor of Metalloproteinase 3 (TIMP3)*  (MIM *188826) | TIMP3 is a marker of mature dendritic cells. Inhibits angiogenesis and cell proliferation as well as induces apoptosis. Potential biomarker of cancer. | [22,27,28] |
| *Insulin-Like Growth Factor-Binding Protein 3 (IGFBP3)*  (MIM *146732) | IGFBP3 affects cell proliferation, migration, and differentiation. Depending on cellular signals, it can stimulate DNA repair and cell growth, or induce apoptosis. Influences the stemness maintenance of endometrium-derived mesenchymal stem cells. | [29,30] |
| *Lim and Calponin Homology Domains-Containing Protein 1 (LIMCH1)*  (MIM *617750) | A negative regulator of cell motility. Potential biomarker for the treatment of cervical cancer. In addition, LIMCH1 is associated with breast cancer. | [31,32] |
| *ZNFX1 Antisense RNA 1 (ZFAS1)* | It affects follicular development and the differentiation of epithelial cells in the mammary gland. Undergoes the above expression in tumor tissues. Associated with progression in colon cancer. | [33,34] |
| *Placenta-Specific Gene 9 (PLAC9)*  (MIM *612857) | Present in the human placenta, fat tissue, prostate, lung, or colon. The elevated expression may be associated with pathological changes in the lungs. | [35,36] |
| *Inhibin, Beta A (INHBA)*  (MIM *147290) | Associated with follicle activation in women and follicle-stimulating hormone secretion in men. Elevated expression levels are present in gastric, esophageal, ovarian, or prostate cancer. | [37,38] |
| *Fibronectin Type III Domain-Containing Protein 1 (FNDC1)*  (MIM * 609991) | FNDC1 presence is detected in the kidneys, thyroid, heart, or digestive system. It plays an important role in the angiogenesis and apoptosis of cardiac cells. Abnormal expression is found in the stomach, prostate, or breast cancer. | [39,40] |
| *Dickopf Wnt Signaling Pathway Inhibitor 1 (DKK1)*  (MIM *605189) | Wnt pathway inhibitor. Expressed in bone, spleen, prostate, colon and placenta. Associated with embryonic development and tumorigenic processes. | [41,42] |
| *Transgelin (TAGLN)*  (MIM *600818) | Associated with smooth muscle differentiation. Interacts with stress fibers in MSCs. Has tumor suppressor effects in some types of cancer. Mainly present in endometrium, prostate, bladder as well as adipose tissue. | [43,44] |
| *Ribosomal Protein S8 (RPS8)*  (MIM *600357) | Encodes a protein that is a component of the small subunit of the 40S ribosome. It is not tissue specific. Increased expression in the presence of tumors and colon polyps. | [45,46] |
| *RNA-Binding Motif Protein 3 (RBM3)*  (MIM *300027) | The elevated expression under hypoxia and cold conditions. Present in highly proliferative tissue. Associated with the proliferation of tumor cells, fibroblasts, and neuronal stem cells. | [47,48] |
| *Keratin 18, Type I (KRT18)*  (MIM *148070) | KRT18 plays a protective role for epithelial cells and replenishes the tissue as intermediate fibers. It provides mechanical resistance to cells and mediates the processes of apoptosis and cell growth. Abundantly expressed in colon, small intestine, liver and stomach. Serves as a biomarker and regulator in many neovascular cancers including breast cancer, hepatocellular carcinoma, gastric and colorectal cancer. | [49,50] |
| *Insulin-Like Growth Factor-Binding Protein 7 (IGFBP7)*  (MIM *602867) | Strongly associated with endothelial cells. Expressed in organs of the gastrointestinal tract and in the brain, spleen, or kidneys. Acts as an oncogene or tumor suppressor gene in various types of cancer. | [51,52] |
| *Matrix Gamma- Carboxyglutamic Acid (MGP)*  (MIM *154870) | Mesenchymal gene, at first isolated from bone tissue. Also present in kidney, lung or cartilage. Overexpressed in skin, breast, and ovarian cancers. Inhibits calcification of arteries and cartilage. | [53,54] |
| *Myosin, Heavy Chain 10, Nonmuscle (MYH10)*  (MIM *160776) | It is part of nonmuscle myosin II. Associated with morphogenesis, cytokinesis, adhesion, cell polarity and motility, transport of organelles. Present in lung, placenta, brain or adipocytes. | [55,56] |
| *Secreted Frizzled-Related Protein 4 (SFRP4)*  (MIM *606570) | Wnt pathway antagonist. Overexpression of the gene is implicated in the pathogenesis of obesity, aging and diabetic complications. In contrast, expression in the myocardium has been linked to apoptosis. | [57,58] |
| *Collagen, Type IV, Alpha-1 (COL4A1)*  (MIM *120130) | A component of the basement membrane, it interacts with other ECM proteins. Expression of this gene is present in endothelial, epithelial, hepatic cells and fibroblasts. Elevated levels seen in urinary bladder cancer and breast cancer. | [59,60] |
| *Ubiquitin Carboxyl-Terminal Esterase L1 (UCHL1)*  (MIM *191342) | It has specific expressions for neurons and cells of the neuroendocrine system. It is strongly expressed in breast cancer. Exhibits ubiquitin hydrolase and ligase activity, associated with proteasomal protein degradation. | [61,62] |
| *Actin, Alpha-2, Smooth Muscle, Aorta (ACTA2)*  (MIM *102620) | ACTA2 is involved in maintaining normal blood pressure and vascular contractility. Present in the endometrium, bladder, prostate and esophagus. | [63,64] |
| *Coagulation Factor II Receptor (F2R)*  (MIM *187930) | Associated with angiogenesis, platelet mobilization, cell proliferation and apoptosis. Modulates the endothelial lining. Expressed in spleen, gallbladder, skin as well as lungs | [65,66] |
| *Insulin-Like Growth Factor-Binding Protein 5 (IGFBP5)*  (MIM *146734) | IGF5 expression is present in bones, lungs, muscles, ovaries and kidneys. It is involved in the response to inflammation, fibrosis, migration and cell proliferation. Overexpression of the gene evident in diabetic complications. | [67,68] |
| *Coiled-Coil Domain-Containing Protein 80 (CCDC80)*  (MIM *608298) | Present in adipocytes and preadipocytes. Protein activity is associated with glycosaminoglycan binding, cell adhesion and response to bacteria. Function is also linked to obesity. | [69,70] |
| *Keratin 7, Type II (KRT7)*  (MIM *148059) | KRT7 is expressed in epithelial cells of internal organs and glandular vessels. It is found in large quantities in the thyroid, lungs or bladder. Altered expression in cancers of the colon, esophagus as well as the cervix. | [71,72] |
| *Secreted Protein, Acidic, Cysteine-Rich (SPARC)*  (MIM *182120) | Bone matrix protein. Associated with bone mineralization, proliferation, differentiation, migration, and angiogenesis. Gene overexpression in gastrointestinal cancers, breast cancer, glioblastoma. | [73,74] |
| *Superoxide Dismutase 3 (SOD3)*  (MIM *185490) | Associated with the extracellular matrix. It has antioxidant as well as prooxidant properties, which involves controlling redox-dependent processes. | [75,76] |
| *Tissue Inhibitor of Metalloproteinase 1 (TIMP1)*  (MIM *305370) | Inhibits the activity of extracellular matrix proteins. Affects cell proliferation and apoptosis. Increased expression in gastric and pancreatic cancer. | [77,78] |
| *Cadherin 2 (CDH2)*  (MIM *114020) | Linked to many physiological processes such as bone composition, cartilage formation, cell adhesion or nervous system development (neurulation, maintenance of synapses, neuronal migration). | [79,80] |
| *Prostaglandin I2 Synthase (PTGIS)*  (MIM *601699) | Present in the tonsils, bladder, heart or lungs in the areas of plasma membranes, nuclear membranes and endoplasmic reticulum. Essential in the vascular system, it regulates vasodilation, inhibits platelet aggregation and cell growth. | [81,82] |
| *Serpin Peptidase Inhibitor, Clade E, Member 2 (SERPINE2)*  (MIM *177010) | Found in the extracellular matrix of endothelial cells, macrophages, fibroblasts or chondrocytes. Regulates the blood clotting system and proteolysis. | [83,84] |
| *Matrix Metalloproteinase 1 (MMP1)*  (MIM *120353) | Involved in degrading ECM components and the basement membrane. Engaged in physiological processes (tissue reconstruction, reproduction, embryogenesis) and pathological ones (inflammation, cardiovascular disease, metastasis). Increased expression in breast, lung, and bladder cancers. | [85,86] |
| *Matrix Metalloproteinase 3 (MMP3)*  (MIM *185250) | Digests various components of the ECM, associated with fibrinolysis, cell differentiation, ossification of cartilage, wound healing, and the development of atherosclerosis. | [87,88] |
| *Superoxide Dismutase 2 (SOD2)*  (MIM *147460) | Mediates regulation of oxidative stress, metabolism and inflammation. Present in mitochondria of appendix, liver, bone marrow, bladder, among others. | [89,90] |
| *S100 Calcium-Binding Protein A4 (S100A4)*  (MIM *114210) | Involved in differentiation, proliferation, cell motility, angiogenesis and cytokine activity. Present in neutrophils, macrophages, T lymphocytes, lung cells, bone marrow cells, appendix cells and adipose. | [91,92] |
| *Interferon-Alpha-Inducible Protein 6 (IFI6)*  (MIM *147572) | Regulates apoptotic activity in mitochondria and cell proliferation. It also has functions related to the immune system. Present in breast and gastric tumors. | [93,94] |
| *Adrenomedullin (ADM)*  (MIM *103275) | Function related to maintaining normal blood pressure, dilating blood vessels. Expressed in the tissues of the cardiovascular system, kidney and lung. Increased expression observed in cardiovascular disease and renal insufficiency. | [95,96] |
| *Tumor Necrosis Factor Receptor Superfamily, Member 11B (TNFRSF11B)*  (MIM *602643) | Associated with bone resorption through negative modulation of osteoclast formation. Supposed to act as a regulator in the cardiovascular system and prevent the arrangement of calcium deposits in the arteries | [97,98] |
| *Cathepsin K (CTSK)*  (MIM *601105) | Present in osteoclasts and chondroclasts, associated with bone resorption and extracellular matrix degradation. Expression is also prominent in breast cancer. | [99,100] |
| *Chemokine, CXC Motif, Ligand 12 (CXCL12)*  (MIM *600835) | Important factor in embryonic development, hematopoiesis, creation of new blood vessels and inflammation. Also associated with cancer growth. Expressed by bone marrow stromal cells and endothelial cells in the brain. | [101,102] |
| *Major Histocompatibility Complex, Class I, B (HLA-B)*  (MIM *142830) | A part of HLA class I, associated with immune response. Expression is seen in spleen, lymph node, lung, intestine, skin. | [103,104] |
| *Ubiquitin-Like Modifier (ISG15)*  (MIM *147571) | Responsible for chemotactic activity against neutrophils, intercellular signaling and antiviral response. Gene expression present in salivary glands, bone marrow, spleen. | [105,106] |
| *Membrane Metalloendopeptidase (MME)*  (MIM *120520) | It has functions related to cell proliferation and migration. Its presence has been detected in intestinal, lung, liver, kidney and brain cells. | [107,108] |
| *RHO Family GTPase 3 (RND3)*  (MIM *602924) | It has functions related to cell migration, proliferation and modulation of the actin cytoskeleton. Furthermore, involved in the development of cardiovascular diseases and cancers (including gastric cancer). | [109,110] |
| *Dermatopontin (DPT)*  (MIM *125597) | Associated with wound healing through increased adhesion of fibroblasts and keratinocytes. Involved in cell interactions with the extracellular matrix. Gene expression detected in adipose tissue, heart, intestine. | [111,112] |
| *C-Type Lectin Domain Family 2, Member B (CLEC2B)*  (MIM *603242) | Responsible for the activation of lymphocytes. Relieved to plasma following platelet mobilization. Highly expressed in skin, spleen and bone marrow. | [113,114] |
| *Cytochrome P450, Family 7, Subfamily B, Polypeptide 1 (CYP7B1)*  (MIM *603711) | Associated with the alternative pathway of bile acid synthesis. Involved in the metabolism of sex hormones. Expressed in the liver, brain, thyroid gland. | [115,116] |
| *Twist Family bHLH Transcription Factor 2 (TWIST2)*  (MIM *607556) | It participates in embryogenesis and inhibits the maturation of muscle and bone cells. Expression of the gene has been detected in adipose tissue, skin, and prostate. | [117,118] |
| *Chemokine, CC Motif, Ligand 2 (CCL2)*  (MIM *158105) | Expressed by fibroblasts, astrocytes, endothelial cells, epithelial cells and smooth muscle cells. Participates in the body's defense and repair processes by attracting cells to affected areas. Overexpression is detected in cancers of the breast, esophagus, stomach, colon, ovary. | [119,120] |
| *MT-RNR2 Like 8 (MTRNR2L8)* | Neuroprotective factor, inhibit apoptosis and inflammation. Associated with stress resistance. Present in both the cytoplasmic and extracellular regions. | [121,122] |

**Supp. Table S12.** List of the top 30 marker genes differentiating between fibroblasts and SASC/VASC.

| **Gene Name** | **FIBRO vs SASC Average log2 fold change** | **Fraction in FIBRO** | **Fraction in SASC** | **P-value (adjusted) p-value SASC** | **FIBRO vs VASC Average log2 fold change** | **Fraction in FIBRO** | **Fraction in VASC** | **P-value (adjusted) p-value VASC** | **Zych et al. 2014 [5]** | **Abreu de Melo et al. 2021 [6]** |
| --- | --- | --- | --- | --- | --- | --- | --- | --- | --- | --- |
| **POSITIVE MARKERS** | | | | | | | | | | |
| *MMP1* | 4,486483 | 0,837 | 0,025 | 0 (0) | 4,505637 | 0,837 | 0,027 | 0 (0) |  | 46,672 |
| *MMP3* | 3,870789 | 0,838 | 0,134 | 0 (0) | 3,945995 | 0,838 | 0,055 | 0 (0) | 5.259032678 | 7,935 |
| *S100A4* | 2,947444 | 0,921 | 0,664 | 0 (0) | 2,863632 | 0,921 | 0,575 | 0 (0) | 2.673123397 | 28,086 |
| *IFI6* | 2,585523 | 0,839 | 0,547 | 0 (0) | 2,56937 | 0,839 | 0,464 | 0 (0) |  | 23,083 |
| *CXCL1* | 2,352146 | 0,451 | 0,008 | 0 (0) | 2,34605 | 0,451 | 0,014 | 0 (0) | 3.512696982 |  |
| *ISG15* | 1,837746 | 0,825 | 0,846 | 0 (0) | 1,861943 | 0,825 | 0,746 | 0 (0) |  | 16,882 |
| *CTSK* | 1,824697 | 0,942 | 0,883 | 0 (0) | 2,504377 | 0,942 | 0,716 | 0 (0) |  | 29,446 |
| *CYP7B1* | 1,596877 | 0,596 | 0,173 | 0 (0) | 1,609551 | 0,596 | 0,131 | 0 (0) |  | 22,359 |
| *LY6E* | 1,539429 | 0,865 | 0,95 | 0 (0) | 1,542205 | 0,865 | 0,892 | 0 (0) |  | 15,997 |
| *TMEM176B* | 1,529808 | 0,497 | 0,088 | 0 (0) | 1,588556 | 0,497 | 0,02 | 0 (0) |  | 57,182 |
| **NEGATIVE MARKERS** | | | | | | | | | | |
| *CCDC80* | -1,59409 | 0,832 | 0,994 | 0 (0) | -2,00652 | 0,832 | 0,993 | 0 (0) | -2.526992064 | -11,116 |
| *COL4A1* | -1,60375 | 0,444 | 0,912 | 0 (0) | -2,13569 | 0,444 | 0,943 | 0 (0) | N/S | -30,782 |
| *CLIC3* | -1,61727 | 0,166 | 0,833 | 0 (0) | -1,5986 | 0,166 | 0,782 | 0 (0) | -4.004096448 | N/S |
| *SPARC* | -1,70121 | 0,886 | 0,996 | 0 (0) | -2,03238 | 0,886 | 0,998 | 0 (0) | -3.27378479 | -15,371 |
| *EFEMP1* | -1,7381 | 0,2 | 0,908 | 0 (0) | -1,78376 | 0,2 | 0,894 | 0 (0) | -3.323299768 | -26,327 |
| *CDH2* | -1,80326 | 0,162 | 0,942 | 0 (0) | -2,05581 | 0,162 | 0,941 | 0 (0) | -4.222925846 | -31,613 |
| *TPM1* | -1,83299 | 0,894 | 0,999 | 0 (0) | -2,07155 | 0,894 | 0,999 | 0 (0) | -2.19301827 | N/S |
| *KRT7* | -1,84339 | 0,133 | 0,884 | 0 (0) | -2,14138 | 0,133 | 0,842 | 0 (0) | -4.386368816 | -23,696 |
| *VCAN* | -2,00011 | 0,691 | 0,984 | 0 (0) | -2,0722 | 0,691 | 0,972 | 0 (0) | -3.202347268 | -21,481 |
| *ACTA2* | -2,04002 | 0,512 | 0,874 | 0 (0) | -2,41084 | 0,512 | 0,896 | 0 (0) | -3.273133043 | -20,362 |
| *IGFBP7* | -2,09029 | 0,79 | 0,994 | 0 (0) | -2,71296 | 0,79 | 0,987 | 0 (0) | -3.825338238 | N/S |
| *INHBA* | -2,10413 | 0,307 | 0,937 | 0 (0) | -2,08054 | 0,307 | 0,917 | 0 (0) | -3.916208979 | -24,108 |
| *TIMP3* | -2,15144 | 0,641 | 0,981 | 0 (0) | -2,05469 | 0,641 | 0,957 | 0 (0) | -4.48098573 | N/S |
| *IGFBP5* | -2,31262 | 0,18 | 0,775 | 0 (0) | -2,58867 | 0,18 | 0,802 | 0 (0) | -2.553521471 | -38,544 |
| *POSTN* | -2,48366 | 0,767 | 0,98 | 0 (0) | -2,44336 | 0,767 | 0,907 | 0 (0) | -4.277563864 | -42,901 |
| *TAGLN* | -2,65058 | 0,714 | 0,994 | 0 (0) | -2,77994 | 0,714 | 0,989 | 0 (0) | -2.623927376 | -27,861 |
| *MGP* | -2,68571 | 0,054 | 0,765 | 0 (0) | -3,13864 | 0,054 | 0,781 | 0 (0) | N/S | -30,292 |
| *PI16* | -2,96732 | 0,004 | 0,624 | 0 (0) | -1,79624 | 0,004 | 0,398 | 0 (0) | -4.298649546 | -49,878 |
| *MFAP5* | -3,20671 | 0,199 | 0,989 | 0 (0) | -3,23205 | 0,199 | 0,973 | 0 (0) | -3.921596288 | -3,697 |
| *COMP* | -3,72632 | 0,22 | 0,954 | 0 (0) | -2,9054 | 0,22 | 0,853 | 0 (0) | -2.449051665 | -20,326 |

**Gene name**: Subgroup of 10 upregulated and 20 downregulated genes in fibroblasts, each showing at least a 1.5 log2 fold change (adjusted p-value < 0.001), consistent expression in both SASCs and VASCs, and concordance across at least two independent studies (including the current study). This subgroup of 30 genes, meeting the specified criteria, was selected from an original set of 1,509 genes differentially expressed in fibroblasts compared to SASC or VASC (adjusted p-value < 0.05), based on scRNA-seq results (Supp. Table S10 and Figure 3). **FIBRO vs SASC Average log2 fold change** and **FIBRO vs VASC Average log2 fold change**: Log2-transformed mean fold changes in gene expression from single-cell RNA sequencing between fibroblasts and SASC or VASC, respectively; **Fraction in FIBRO/SACS/VASC**: fraction of cells expressing the specified gene in each cell type. **Zych et al. 2014 [5] and Abreu de Melo et al. 2021 [6]**: expression values of the corresponding genes from Zych et al. 2014 (PMID: 25068904) and Abreu de Melo et al. 2021 (PMID: 33389760); **NS**: not specified.

**Supplementary Table S13.** Results of confirmatory quantitative PCR analysis of selected AD-MSC and fibroblast markers.

| **Gene** | **SASC_1 Mean ±SD** | **SASC_4 Mean ±SD** | **VASC_2 Mean ±SD** | **VASC_5 Mean ±SD** | **(SV)ASC Mean** | **FIBRO_3 Mean ±SD** | **FIBRO_6 Mean ±SD** | **FIBRO Mean** | **FIBRO Mean/ (SV)ASC Mean** | **qPCR Log_2_ Fold Change** | **scRNA-seq Mean Log_2_ Fold Change SASC** | **scRNA-seq Mean Log_2_ Fold Change VASC** |
| --- | --- | --- | --- | --- | --- | --- | --- | --- | --- | --- | --- | --- |
| ***CXCL1*** | 0,12± 0,02 | 0,38± 0,00 | 0,80±0,02 | 0,37±0,00 | **0,42** | 16,45±0,14 | 31,65±1,10 | **24,05** | **57,80** | **5,85** | 2,35 | 2,35 |
| ***S100A4*** | 0,38±0,00 | 0,05± 0,00 | 0,42±0,01 | 0,10±0,00 | **0,24** | 2,30±0,00 | 1,67±0,02 | **1,98** | **8,36** | **3,06** | 2,95 | 2,86 |
| ***MMP1*** | 0,60±0,00 | 0,04±0,00 | 0,78±0,01 | 0,05±0,00 | **0,36** | 13,56±0,14 | 22,58±0,31 | **18,07** | **49,53** | **5,63** | 4,49 | 4,51 |
| ***MMP3*** | 2,25±0,02 | 0,26± 0,00 | 0,02±0,00 | 0,30±0,01 | **0,71** | 18,47±0,13 | 48,60±0,17 | **33,53** | **47,37** | **5,57** | 3,87 | 3,95 |
| ***COMP*** | 14,20±0,12 | 27,49±1,05 | 7,28±0,03 | 25,43±0,26 | **18,60** | 0,03±0,00 | 0,29±0,01 | **0,16** | **0,01** | **-6,87** | -3,73 | -2,91 |
| ***IGFBP5*** | 0,35±0,01 | 0,71±0,01 | 0,60±0,02 | 0,53±0,00 | **0,55** | 0,01±0,00 | 0,08±0,01 | **0,05** | **0,09** | **-3,49** | -2,31 | -2,59 |
| ***PI16*** | 2,63±0,01 | 0,11±0,00 | 0,49±0,01 | 0,15±0,01 | **0,84** | 0,00±0,00 | 0,00±0,00 | **0,00** | **0,00** | **-9,88** | -2,97 | -1,80 |

**Gene:** genes with contrasting expression differences between AD-MSCs and fibroblasts, selected for confirmatory qPCR from the set shown in Supplementary Table S12. The relative expression levels of these seven genes are sufficient to distinguish between AD-MSCs and fibroblasts. **SACS_1_4, VASC_2_5, FIBRO_3_6 Mean ±SD**: expression mean and standard deviation between technical replicate qPCR experiments; **SV(ASC) Mean:** stromal and vascular adipose derived mesenchymal stromal cells expression mean between biological replicates; **FIBRO Mean:** fibroblasts expression mean between biological replicates; **FIBRO Mean/(SV)ASC Mean***:* ratio of mean expression between fibroblats and AD-MSCs; **qPCR Log2 Fold Change:** Log_2_-transformed fold change in gene expression between fibroblasts and AD-MSCs; **scRNA-seq Mean Log2** **Fold Change SASC** and **scRNA-seq Mean Log2 Fold Change VASC:** Log_2_-transformed mean fold changes in gene expression from single-cell RNA sequencing between fibroblasts and SASC or VASC, respectively (Supplementary Table S12). SASC_1, VASC_2, FIBRO_3 were derived from donor #1. SASC_4, VASC_5, FIBRO_6 were derived from donor #2. The corresponding technical description of qPCR assay is provided in Materials and Method under "Quantitative PCR analysis of selected AD-MSCs and fibroblasts markers" subsection; qPCR primer sequences are provided in Supplementary Table S1.

**
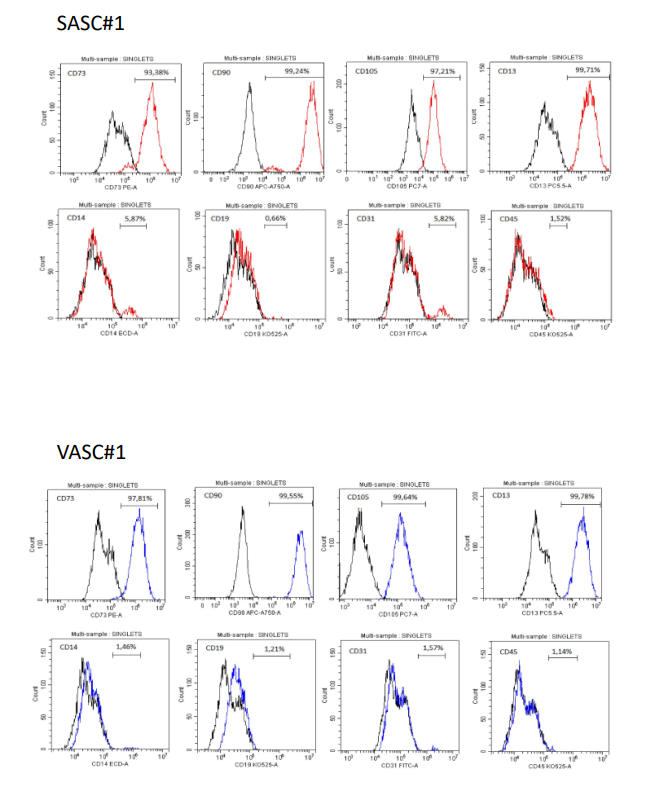
**

**
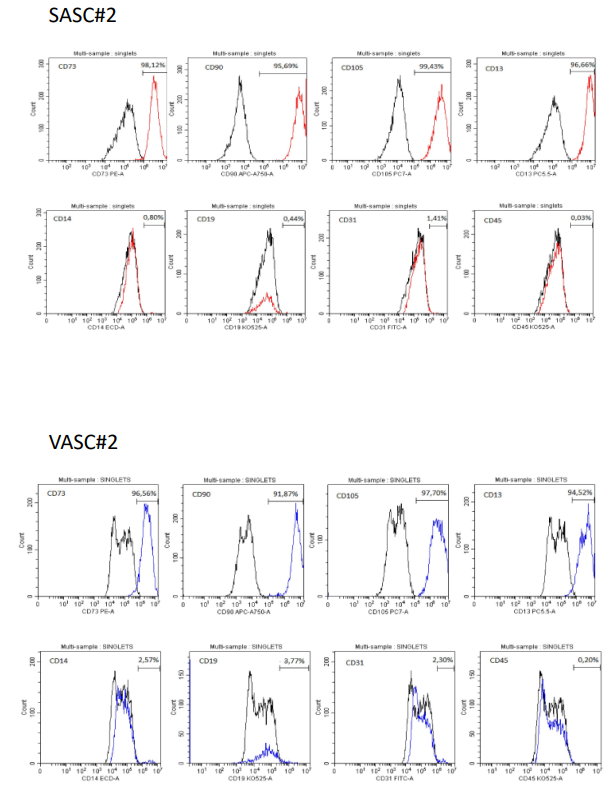

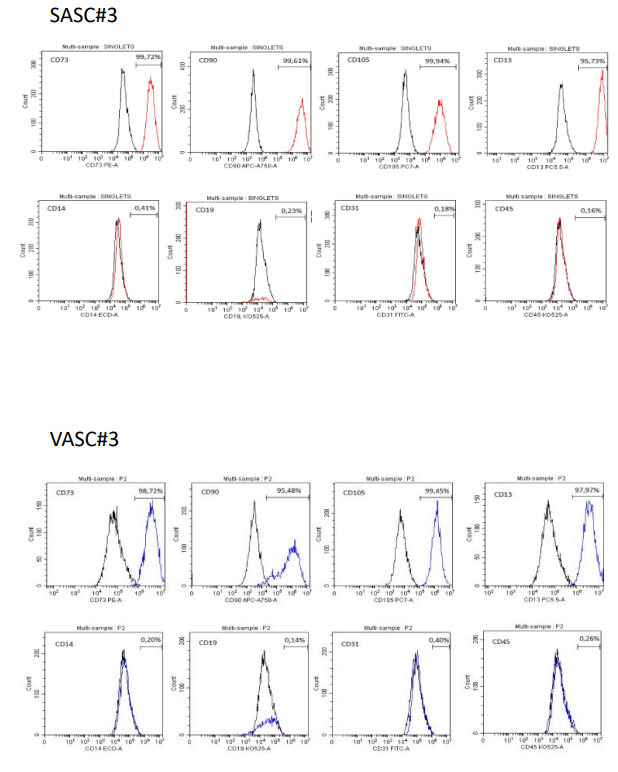

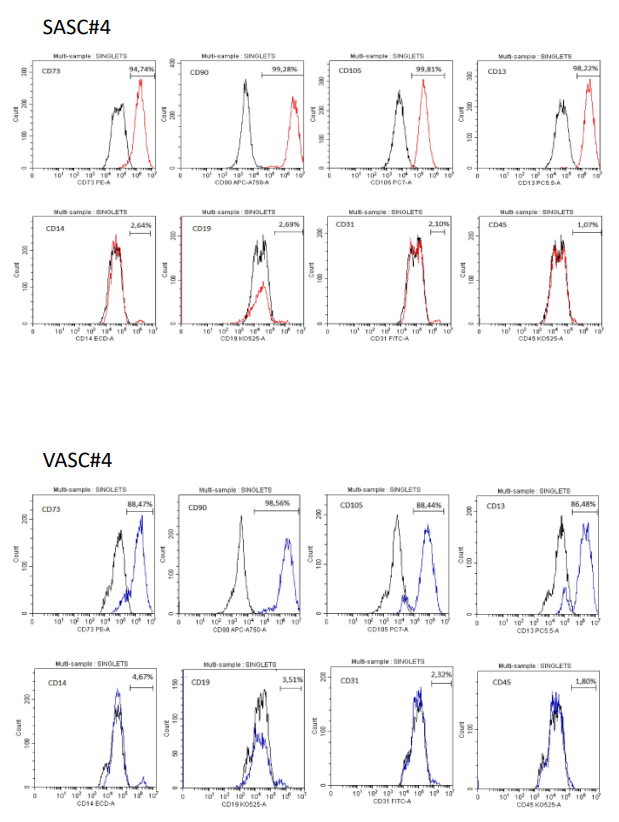
**

**Supp. Figure S1.** Flow cytometry analysis of subcutaneous (SASC) and visceral (VASC) adipose derived mesenchymal stromal cells.

At passage 3, SASC and VASC were evaluated for the expression of positive stem/stromal cell surface markers (CD73, CD90, CD105) and negative markers (CD14, CD19, CD45). Additional positive (CD13) and negative (CD31) markers were also assessed. Cell viability was determined using DAPI staining. The descriptions of the panels correspond to SASC and VASC samples obtained from four unrelated individuals, where SASC#1 and VASC#1 represent samples collected from individual #1.

**
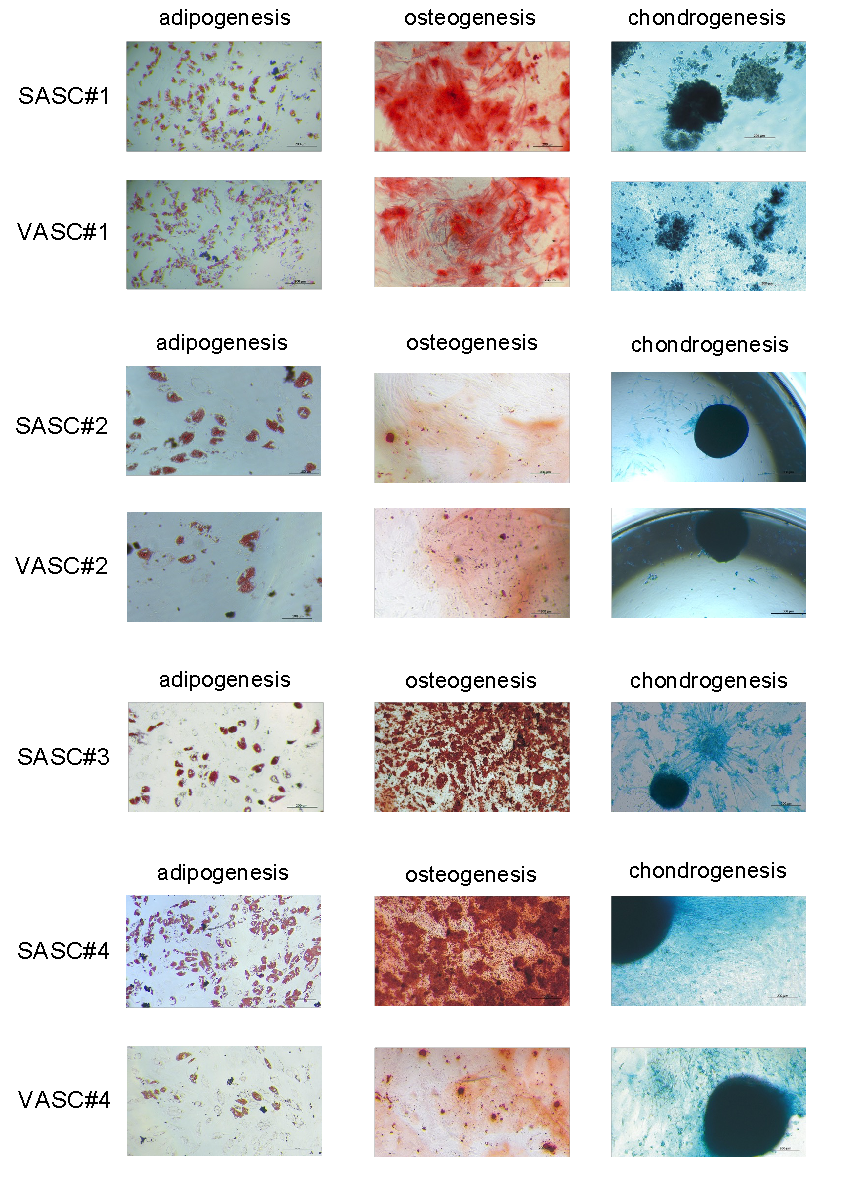
 Supp. Figure S2.** Differentiation capacity of SASC and VASC subcutaneous collected from four unrelated individuals.

The VASC#3 sample was not differentiated due to insufficient source material.


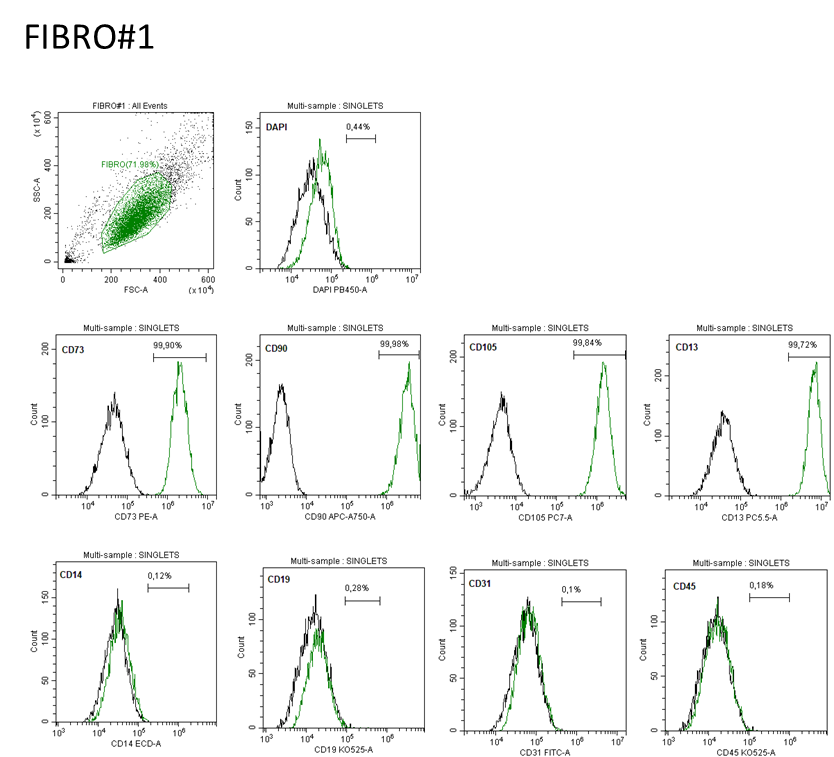


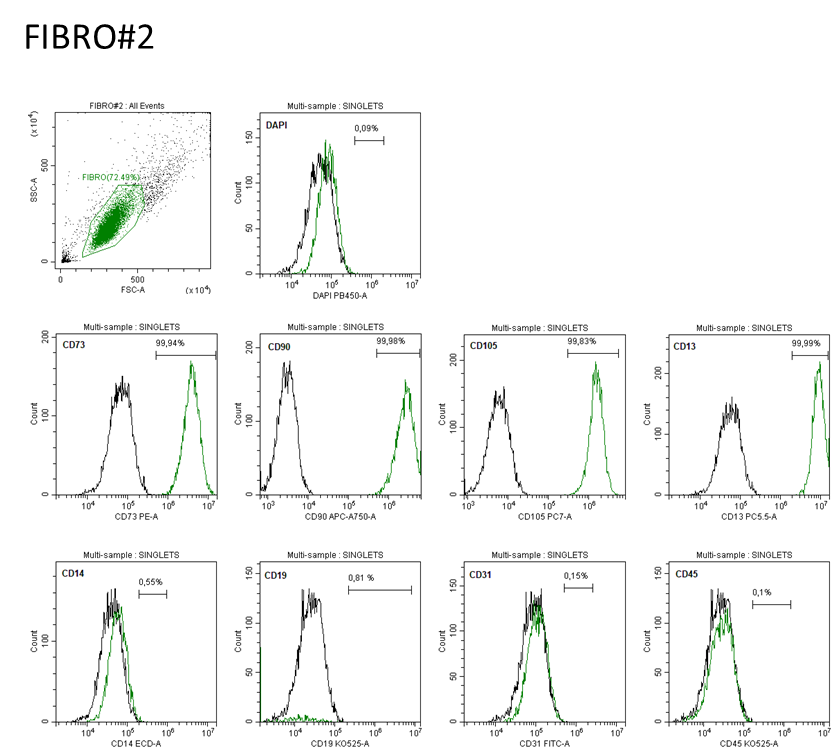


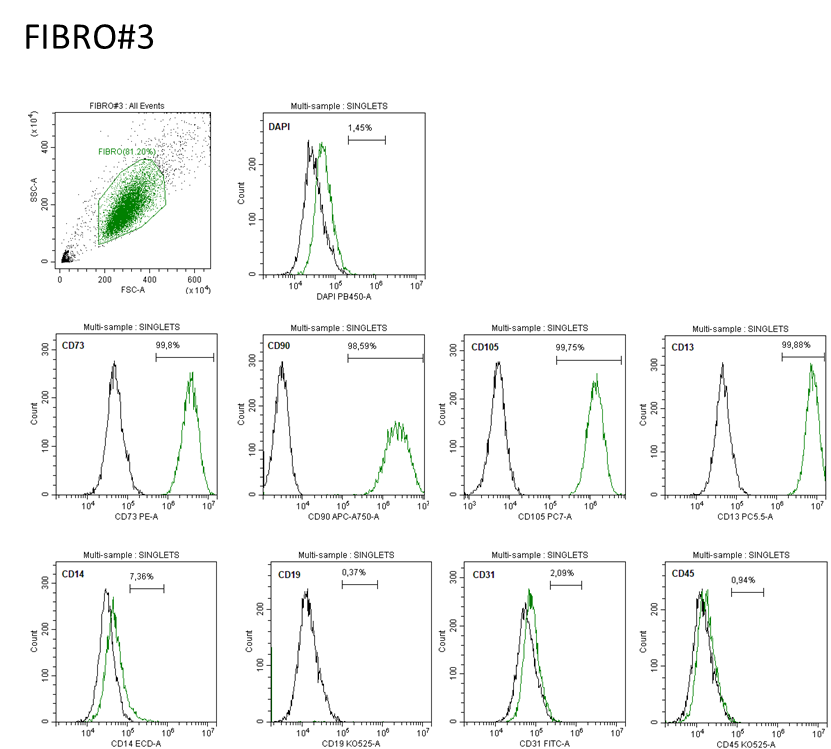


**Supp. Figure S3.** Flow cytometry analysis of dermal fibroblasts.

At passage 3, dermal fibroblasts (FIBRO) were evaluated for the expression of positive stem/stromal cell surface markers (CD73, CD90, CD105, CD13, CD14, CD19, CD45, CD31). The description of the panels correspond to FIBRO samples collected from four unrelated individuals (FIBRO#1, FIBRO#2, FIBRO#3).


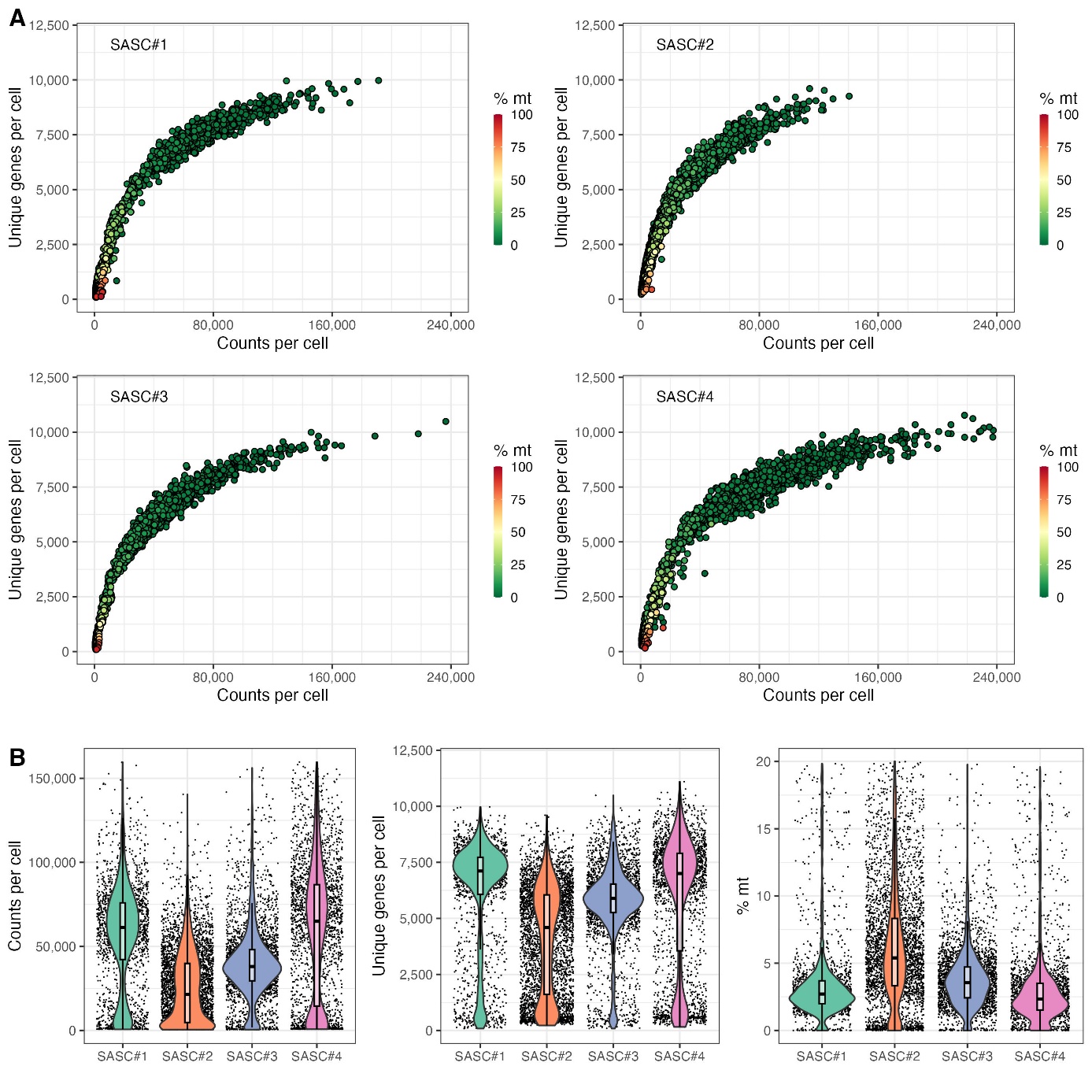


**Supp. Figure S4.** Quality control of single-cell RNA sequencing data of subcutaneous AD-MSCs collected from four individuals (SASC#1-SASC#4).

The plots display the number of detected genes and the percentage of identified mitochondrial transcripts in SASC data (A). Violin plots represent the counts per cell, identified genes, and mitochondrial reads in SASC data (B).


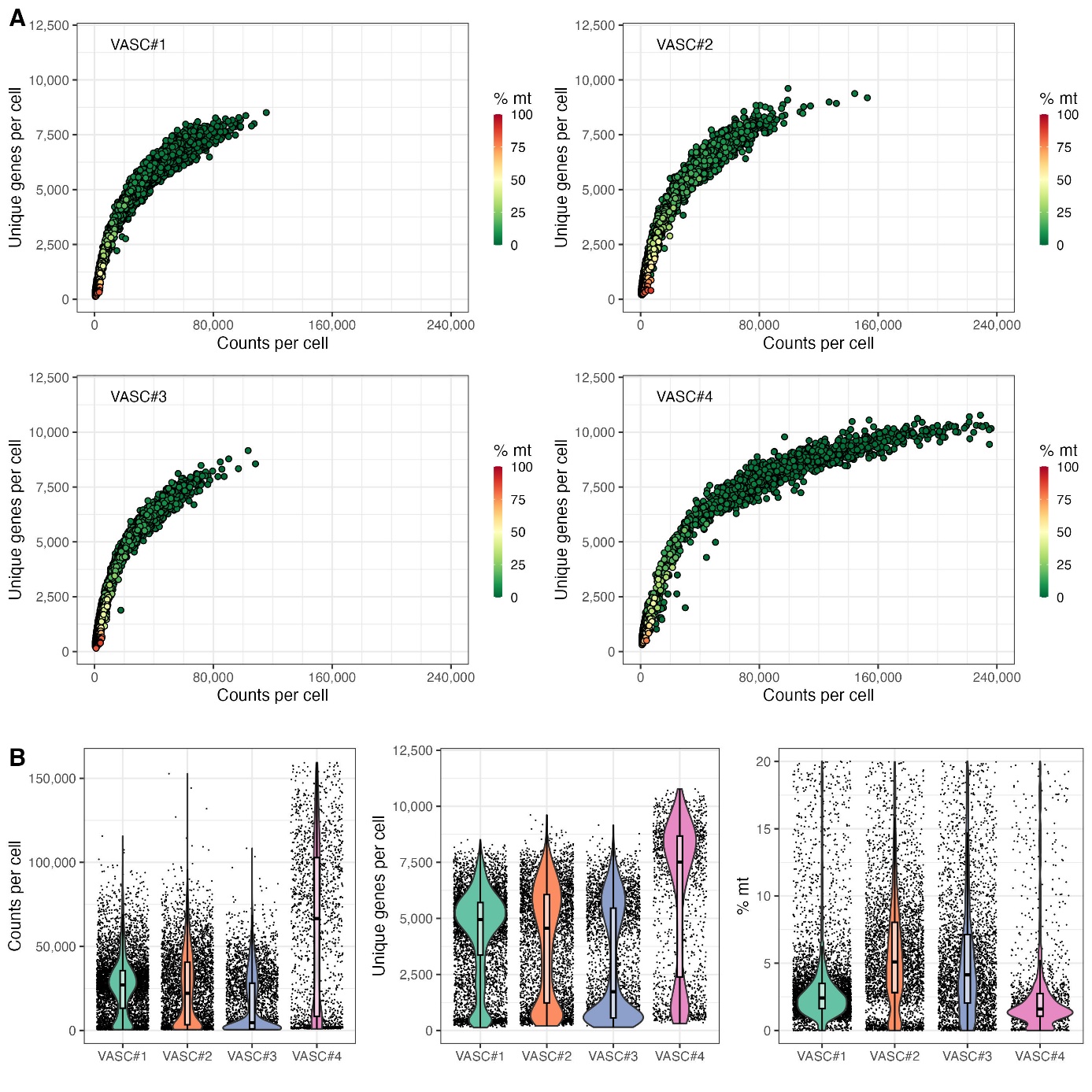


**Supp. Figure S5.** Quality control of single-cell RNA sequencing data of visceral AD-MSCs collected from four individuals (VASC#1-VASC#4).

The plots display the number of detected genes and the percentage of identified mitochondrial transcripts in VASC data (A). Violin plots represent the counts per cell, identified genes, and mitochondrial reads in VASC data (B).


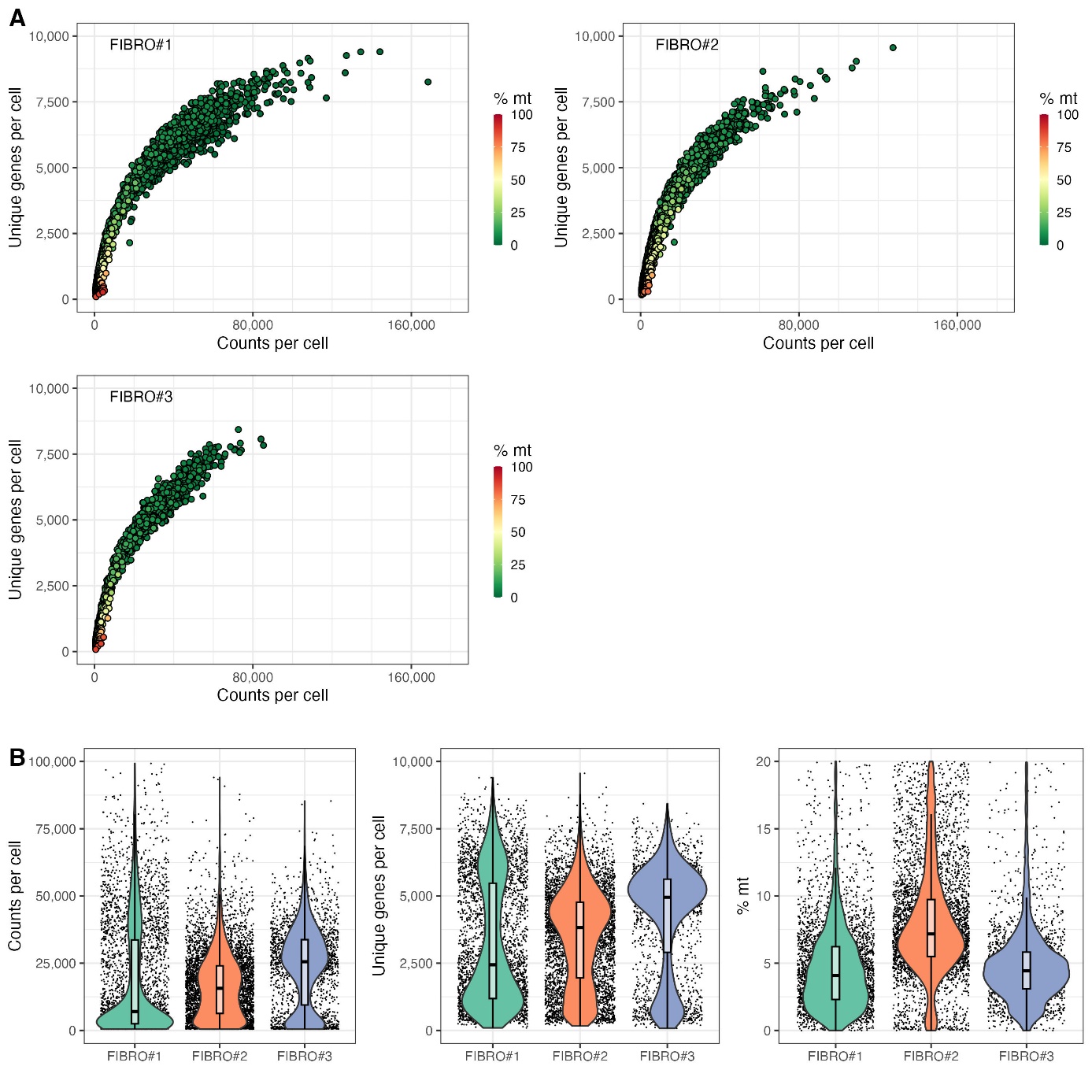


**Supp. Figure S6.** Quality control of single-cell RNA sequencing data of dermal fibroblasts collected from three individuals (FIBRO#1-FIBRO#3).

The plots display the number of detected genes and the percentage of identified mitochondrial transcripts in FIBRO data (A). Violin plots represent the counts per cell, identified genes, and mitochondrial reads in FIBRO data (B).


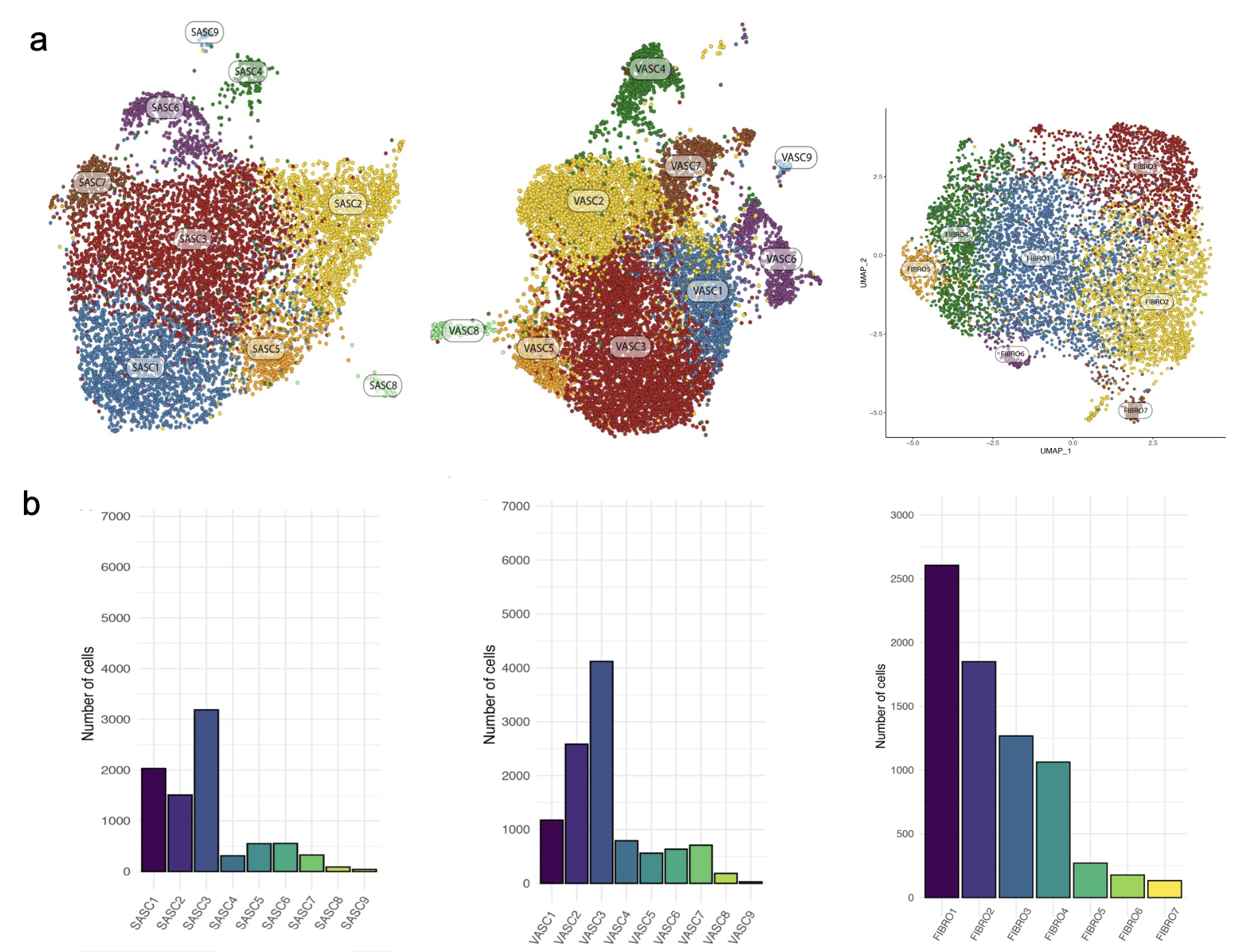


FIBRO

VASC

SASC

**Supp. Figure S7.** Cell distribution and marker expression of SASC, VASC, and FIBRO.

Uniform Manifold Approximation and Projection (UMAP) plots representing the SASC and VASC distribution (A) and the number of cells expressing positive stem/stromal marker genes in SASC (first bar chart) and VASC (second bar chart) clusters, as well as cell distribution and marker expression in fibroblast clusters (third bar chart) (B).


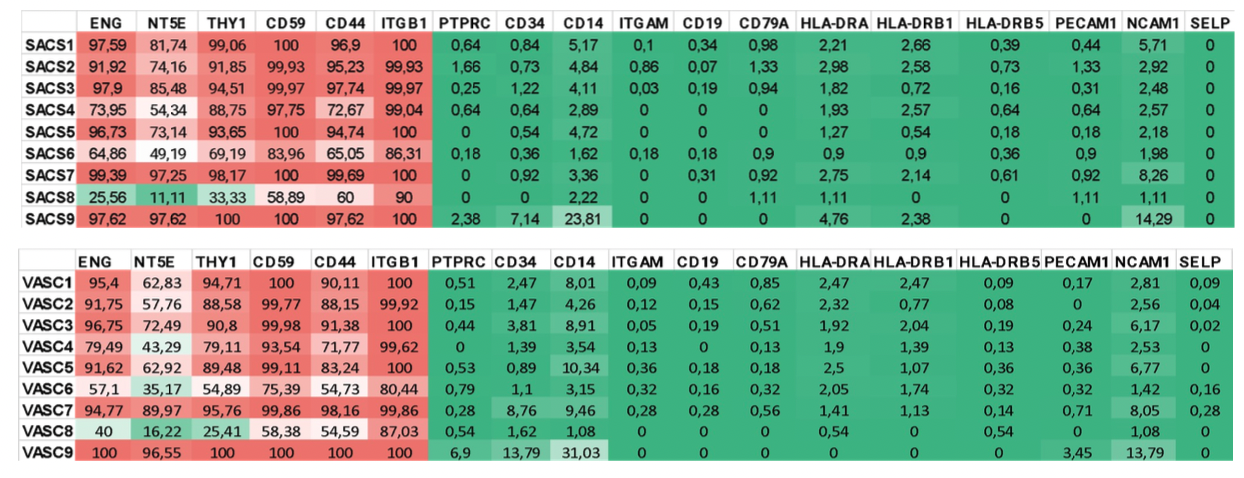


**Supp. Figure S8.** Integrated expression of stem/stromal marker genes in clusters 1-9 of SASC and VASC from the four individuals analyzed in this study.

The values represent the percentage of cells expressing each marker gene based on the list of positive and negative markers recommended by the International Federation for Adipose Therapeutics and Science (IFATS) and the International Society for Cellular Therapy (ISCT) [123–126].

*Positive stem/stromal marker genes:*

*ENG* (MIM *131195) - alternative symbol *CD105; NT5E* (MIM *129190) - alternative symbol *CD73; THY1* (MIM *188230) - alternative symbol *CD90; CD59* (MIM *107271)*; CD44* (MIM *107269); *ITGB1* (MIM *135630) - alternative symbol *CD29.*

*Negative stem/stromal marker genes:*

*PTPRC* (MIM *151460) - alternative symbol *CD45; CD34* (MIM *142230); *CD14* (MIM *158120); *ITGAM* (MIM *120980) - alternative symbol *CD11B; CD19* (MIM *107265); *CD79A* (MIM *112205); *HLA-DRA* (MIM *142860); *HLA-DRB1* (MIM *142857); *HLA-DRB5* (MIM *604776); *PECAM1* (MIM *173445) - alternative symbol *CD31; NCAM1* (MIM *116930) - alternative symbol *CD56; SELP* (MIM *173610) - alternative symbol *CD62.*


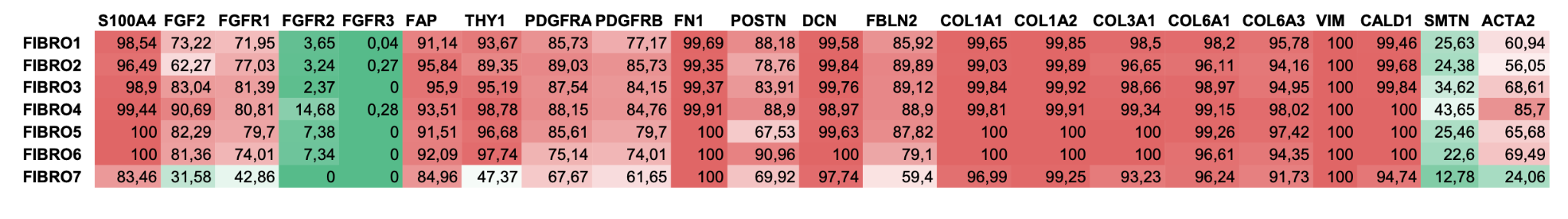
**Supp. Figure S9.** Integrated expression of positive and negative fibroblast marker genes in clusters 1-7 of dermal fibroblasts from the three individuals analyzed in this study.

The values represent the percentage of cells expressing each marker gene based on the list of positive and negative markers described and confirmed in other studies over the years [127–130].

*Positive fibroblast marker genes:*

*S100A4* (MIM *131195) - alternative symbol *CAPL & FSP1; FGF2* (MIM *134920); *FGFR1* (MIM *136350); *FAP* (MIM *600403); *THY1* (MIM *188230) - alternative symbol CD90; *PDGFRA* (MIM *173490); *PDGFRB* (MIM *173410); *FN1* (MIM *135600); *POSTN* (MIM *608777); *DCN* (MIM *125255); *FBLN2* (MIM *135821); *COL1A1* (MIM *120150); *COL1A2* (MIM *120160); *COL3A1* (MIM *120180); *COL6A1* (MIM *120220); *COL6A3* (MIM *120250); *VIM* (MIM *193060); *CALD1* (MIM *114213); *ACTA2* (MIM *102620).

*Negative fibroblast marker genes:*

*FGFR2* (MIM *176943); *FGFR3* (MIM *134934); *SMTN* (MIM *602127).


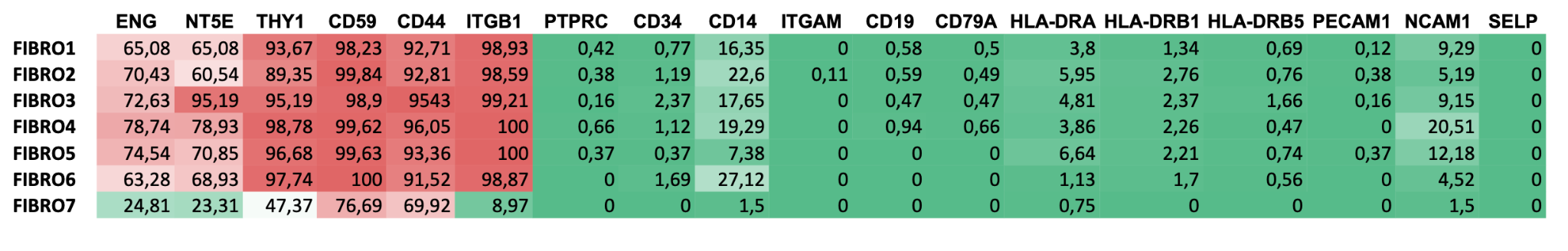
**Supp. Figure S10.** Integrated expression of positive and negative stem/stromal marker genes in clusters 1-7 of dermal fibroblasts from the three individuals analyzed in this study.

The values represent the percentage of cells expressing each marker gene based on the list of positive and negative markers recommended by the International Federation for Adipose Therapeutics and Science (IFATS) and the International Society for Cellular Therapy (ISCT) [123–126].

*Positive stem/stromal marker genes:*

*ENG* (MIM *131195) - alternative symbol *CD105; NT5E* (MIM *129190) - alternative symbol *CD73; THY1* (MIM *188230) - alternative symbol *CD90; CD59* (MIM *107271)*; CD44* (MIM *107269); *ITGB1* (MIM *135630) - alternative symbol *CD29.*

*Negative stem/stromal marker genes:*

*PTPRC* (MIM *151460) - alternative symbol *CD45; CD34* (MIM *142230); *CD14* (MIM *158120); *ITGAM* (MIM *120980) - alternative symbol *CD11B; CD19* (MIM *107265); *CD79A* (MIM *112205); *HLA-DRA* (MIM *142860); *HLA-DRB1* (MIM *142857); *HLA-DRB5* (MIM *604776); *PECAM1* (MIM *173445) - alternative symbol *CD31; NCAM1* (MIM *116930) - alternative symbol *CD56; SELP* (MIM *173610) - alternative symbol *CD62.*

**References**

1. Amin MB, Greene FL, Edge SB, Compton CC, Gershenwald JE, Brookland RK, et al. The Eighth Edition AJCC Cancer Staging Manual: Continuing to build a bridge from a population-based to a more “personalized” approach to cancer staging. CA Cancer J Clin. 2017;67:93–9.

2. Vijay J, Gauthier M-F, Biswell RL, Louiselle DA, Johnston JJ, Cheung WA, et al. Single-cell analysis of human adipose tissue identifies depot- and disease-specific cell types. Nat Metab. 2020;2:97–109.

3. Emont MP, Jacobs C, Essene AL, Pant D, Tenen D, Colleluori G, et al. A single-cell atlas of human and mouse white adipose tissue. Nature. 2022;603:926–33.

4. Qu X, Liu H, Song X, Sun N, Zhong H, Qiu X, et al. Effective degradation of EGFRL858R+T790M mutant proteins by CRBN-based PROTACs through both proteosome and autophagy/lysosome degradation systems. Eur J Med Chem. 2021;218:113328.

5. Zych J, Spangenberg L, Stimamiglio MA, Abud APR, Shigunov P, Marchini FK, et al. Polysome profiling shows the identity of human adipose-derived stromal/stem cells in detail and clearly distinguishes them from dermal fibroblasts. Stem Cells Dev. 2014;23:2791–802.

6. Abreu de Melo MI, da Silva Cunha P, Coutinho de Miranda M, Faraco CCF, Barbosa JL, da Fonseca Ferreira A, et al. Human adipose-derived stromal/stem cells are distinct from dermal fibroblasts as evaluated by biological characterization and RNA sequencing. Cell Biochem Funct. 2021;39:442–54.

6. Amin MB, Greene FL, Edge SB, Compton CC, Gershenwald JE, Brookland RK, et al. The Eighth Edition AJCC Cancer Staging Manual: Continuing to build a bridge from a population-based to a more “personalized” approach to cancer staging. CA Cancer J Clin. 2017;67:93–9.

7. Hazell GGJ, Peachey AMG, Teasdale JE, Sala-Newby GB, Angelini GD, Newby AC, et al. PI16 is a shear stress and inflammation-regulated inhibitor of MMP2. Sci Rep. 2016;6:39553.

8. Regn M, Laggerbauer B, Jentzsch C, Ramanujam D, Ahles A, Sichler S, et al. Peptidase inhibitor 16 is a membrane-tethered regulator of chemerin processing in the myocardium. Journal of Molecular and Cellular Cardiology. 2016;99:57–64.

9. Halász K, Kassner A, Mörgelin M, Heinegård D. COMP acts as a catalyst in collagen fibrillogenesis. J Biol Chem. 2007;282:31166–73.

10. Posey KL, Coustry F, Hecht JT. Cartilage oligomeric matrix protein: COMPopathies and beyond. Matrix Biol. 2018;71–72:161–73.

11. Crisponi L, Buers I, Rutsch F. CRLF1 and CLCF1 in Development, Health and Disease. International Journal of Molecular Sciences. 2022;23:992.

12. Huang H-B, Luo H-T, Wei N-N, Liu M-L, He F, Yang W, et al. Integrative analysis reveals a lineage-specific circular RNA landscape for adipo-osteogenesis of human mesenchymal stem cells. Stem Cell Research & Therapy. 2022;13:106.

13. Wang B, Suen CW, Ma H, Wang Y, Kong L, Qin D, et al. The Roles of H19 in Regulating Inflammation and Aging. Frontiers in Immunology [Internet]. 2020 [cited 2023 Oct 11];11. Available from: https://www.frontiersin.org/articles/10.3389/fimmu.2020.579687

14. Zhou Z, Hossain MS, Liu D. Involvement of the long noncoding RNA H19 in osteogenic differentiation and bone regeneration. Stem Cell Research & Therapy. 2021;12:74.

15. Lescan M, Perl RM, Golombek S, Pilz M, Hann L, Yasmin M, et al. De Novo Synthesis of Elastin by Exogenous Delivery of Synthetic Modified mRNA into Skin and Elastin-Deficient Cells. Molecular Therapy - Nucleic Acids. 2018;11:475–84.

16. Ozsvar J, Yang C, Cain SA, Baldock C, Tarakanova A, Weiss AS. Tropoelastin and Elastin Assembly. Frontiers in Bioengineering and Biotechnology [Internet]. 2021 [cited 2023 Oct 11];9. Available from: https://www.frontiersin.org/articles/10.3389/fbioe.2021.643110

17. Li H, Zhou W, Sun S, Zhang T, Zhang T, Huang H, et al. Microfibrillar-associated protein 5 regulates osteogenic differentiation by modulating the Wnt/β-catenin and AMPK signaling pathways. Molecular Medicine. 2021;27:153.

18. Vaittinen M, Kolehmainen M, Rydén M, Eskelinen M, Wabitsch M, Pihlajamäki J, et al. MFAP5 is related to obesity-associated adipose tissue and extracellular matrix remodeling and inflammation. Obesity. 2015;23:1371–8.

19. Han W, Shi J, Cao J, Dong B, Guan W. <p>Latest Advances of Long Non-Coding RNA SNHG5 in Human Cancers</p>. OTT. 2020;13:6393–403.

20. Li Y, Guo D, Zhao Y, Ren M, Lu G, Wang Y, et al. Long non-coding RNA SNHG5 promotes human hepatocellular carcinoma progression by regulating miR-26a-5p/GSK3β signal pathway. Cell Death Dis. 2018;9:1–15.

21. González-González L, Alonso J. Periostin: A Matricellular Protein With Multiple Functions in Cancer Development and Progression. Frontiers in Oncology [Internet]. 2018 [cited 2023 Oct 11];8. Available from: https://www.frontiersin.org/articles/10.3389/fonc.2018.00225

22. Han L, Gong S, Wang R, Liu S, Wang B, Chen G, et al. Knockdown of POSTN Inhibits Osteogenic Differentiation of Mesenchymal Stem Cells From Patients With Steroid-Induced Osteonecrosis. Frontiers in Cell and Developmental Biology [Internet]. 2020 [cited 2023 Oct 11];8. Available from: https://www.frontiersin.org/articles/10.3389/fcell.2020.606289

23. Zhang F, Zhang Z, Sun D, Dong S, Xu J, Dai F. Periostin: A Downstream Mediator of EphB4-Induced Osteogenic Differentiation of Human Bone Marrow-Derived Mesenchymal Stem Cells. Stem Cells International. 2015;2016.

24. Alfaro MP, Deskins DL, Wallus M, DasGupta J, Davidson JM, Nanney LB, et al. A physiological role for connective tissue growth factor in early wound healing. Lab Invest. 2013;93:81–95.

25. Leguit RJ, Raymakers RAP, Hebeda KM, Goldschmeding R. CCN2 (Cellular Communication Network factor 2) in the bone marrow microenvironment, normal and malignant hematopoiesis. J Cell Commun Signal. 2021;15:25–56.

26. Wu Y-L, Li H-Y, Zhao X-P, Jiao J-Y, Tang D-X, Yan L-J, et al. Mesenchymal stem cell-derived CCN2 promotes the proliferation, migration and invasion of human tongue squamous cell carcinoma cells. Cancer Sci. 2017;108:897–909.

27. Almalki SG, Agrawal DK. Effects of matrix metalloproteinases on the fate of mesenchymal stem cells. Stem Cell Research & Therapy. 2016;7:129.

28. Fan D, Kassiri Z. Biology of Tissue Inhibitor of Metalloproteinase 3 (TIMP3), and Its Therapeutic Implications in Cardiovascular Pathology. Frontiers in Physiology [Internet]. 2020 [cited 2023 Oct 11];11. Available from: https://www.frontiersin.org/articles/10.3389/fphys.2020.00661

29. Ushakov RE, Skvortsova EV, Vitte MA, Vassilieva IO, Shatrova AN, Kotova AV, et al. Chondrogenic differentiation followed IGFBP3 loss in human endometrial mesenchymal stem cells. Biochemical and Biophysical Research Communications. 2020;531:133–9.

30. Varma Shrivastav S, Bhardwaj A, Pathak KA, Shrivastav A. Insulin-Like Growth Factor Binding Protein-3 (IGFBP-3): Unraveling the Role in Mediating IGF-Independent Effects Within the Cell. Frontiers in Cell and Developmental Biology [Internet]. 2020 [cited 2023 Oct 11];8. Available from: https://www.frontiersin.org/articles/10.3389/fcell.2020.00286

31. Halle MK, Sødal M, Forsse D, Engerud H, Woie K, Lura NG, et al. A 10-gene prognostic signature points to LIMCH1 and HLA-DQB1 as important players in aggressive cervical cancer disease. Br J Cancer. 2021;124:1690–8.

32. Tashireva L, Gerashchenko T, Alifanov V, Perelmuter V, Cherdyntseva N. 51P - LIMCH1-related genes demonstrate different invasive potential of morphological structures of breast cancer. Annals of Oncology. 2019;30:vii16.

33. Dong D, Mu Z, Zhao C, Sun M. ZFAS1: a novel tumor-related long non-coding RNA. Cancer Cell International. 2018;18:125.

34. O’Brien SJ, Fiechter C, Burton J, Hallion J, Paas M, Patel A, et al. Long non-coding RNA ZFAS1 is a major regulator of epithelial-mesenchymal transition through miR-200/ZEB1/E-cadherin, vimentin signaling in colon adenocarcinoma. Cell Death Discov. 2021;7:1–14.

35. Galaviz-Hernandez C, Stagg C, de Ridder G, Tanaka TS, Ko MSH, Schlessinger D, et al. Plac8 and Plac9, novel placental-enriched genes identified through microarray analysis. Gene. 2003;309:81–9.

36. Wang H-X, Qin X-H, Shen J, Liu Q-H, Shi Y-B, Xue L. Proteomic Analysis Reveals That Placenta-Specific Protein 9 Inhibits Proliferation and Stimulates Motility of Human Bronchial Epithelial Cells. Frontiers in Oncology [Internet]. 2021 [cited 2023 Oct 11];11. Available from: https://www.frontiersin.org/articles/10.3389/fonc.2021.628480

37. Wu Z, Tang Y, Niu X, Cheng Q. Expression and gene regulation network of INHBA in Head and neck squamous cell carcinoma based on data mining. Sci Rep. 2019;9:14341.

38. Zhao K, Yi Y, Ma Z, Zhang W. INHBA is a Prognostic Biomarker and Correlated With Immune Cell Infiltration in Cervical Cancer. Frontiers in Genetics [Internet]. 2022 [cited 2023 Oct 11];12. Available from: https://www.frontiersin.org/articles/10.3389/fgene.2021.705512

39. Chen L, Liu J, Wang L, Yang X, Jiang Q, Ji F, et al. Up-regulated FNDC1 accelerates stemness and chemoradiation resistance in colorectal cancer cells. Biochemical and Biophysical Research Communications. 2022;602:84–90.

40. Jiang T, Gao W, Lin S, Chen H, Du B, Liu Q, et al. FNDC1 Promotes the Invasiveness of Gastric Cancer via Wnt/β-Catenin Signaling Pathway and Correlates With Peritoneal Metastasis and Prognosis. Frontiers in Oncology [Internet]. 2020 [cited 2023 Oct 11];10. Available from: https://www.frontiersin.org/articles/10.3389/fonc.2020.590492

41. Chu HY, Chen Z, Wang L, Zhang Z-K, Tan X, Liu S, et al. Dickkopf-1: A Promising Target for Cancer Immunotherapy. Frontiers in Immunology [Internet]. 2021 [cited 2023 Oct 11];12. Available from: https://www.frontiersin.org/articles/10.3389/fimmu.2021.658097

42. Niida A, Hiroko T, Kasai M, Furukawa Y, Nakamura Y, Suzuki Y, et al. DKK1, a negative regulator of Wnt signaling, is a target of the β-catenin/TCF pathway. Oncogene. 2004;23:8520–6.

43. Tsuji-Tamura K, Morino-Koga S, Suzuki S, Ogawa M. The canonical smooth muscle cell marker TAGLN is present in endothelial cells and is involved in angiogenesis. Journal of Cell Science. 2021;134:jcs254920.

44. Wei X, Lou H, Zhou D, Jia Y, Li H, Huang Q, et al. TAGLN mediated stiffness-regulated ovarian cancer progression via RhoA/ROCK pathway. Journal of Experimental & Clinical Cancer Research. 2021;40:292.

45. Bi N, Sun Y, Lei S, Zeng Z, Zhang Y, Sun C, et al. Identification of 40S ribosomal protein S8 as a novel biomarker for alcohol‑associated hepatocellular carcinoma using weighted gene co‑expression network analysis. Oncology Reports. 2020;44:611–27.

46. Luan Y, Tang N, Yang J, Liu S, Cheng C, Wang Y, et al. Deficiency of ribosomal proteins reshapes the transcriptional and translational landscape in human cells. Nucleic Acids Res. 2022;50:6601–17.

47. Wellmann S, Truss M, Bruder E, Tornillo L, Zelmer A, Seeger K, et al. The RNA-Binding Protein RBM3 Is Required for Cell Proliferation and Protects Against Serum Deprivation-Induced Cell Death. Pediatr Res. 2010;67:35–41.

48. Yan J, Goerne T, Zelmer A, Guzman R, Kapfhammer JP, Wellmann S, et al. The RNA-Binding Protein RBM3 Promotes Neural Stem Cell (NSC) Proliferation Under Hypoxia. Frontiers in Cell and Developmental Biology [Internet]. 2019 [cited 2023 Oct 11];7. Available from: https://www.frontiersin.org/articles/10.3389/fcell.2019.00288

49. Chen B, Xu X, Lin D, Chen X, Xu Y, Liu X, et al. KRT18 Modulates Alternative Splicing of Genes Involved in Proliferation and Apoptosis Processes in Both Gastric Cancer Cells and Clinical Samples. Frontiers in Genetics [Internet]. 2021 [cited 2023 Oct 11];12. Available from: https://www.frontiersin.org/articles/10.3389/fgene.2021.635429

50. Wang P-B, Chen Y, Ding G-R, Du H-W, Fan H-Y. Keratin 18 induces proliferation, migration, and invasion in gastric cancer via the MAPK signalling pathway. Clinical and Experimental Pharmacology and Physiology. 2021;48:147–56.

51. Jin L, Shen F, Weinfeld M, Sergi C. Insulin Growth Factor Binding Protein 7 (IGFBP7)-Related Cancer and IGFBP3 and IGFBP7 Crosstalk. Frontiers in Oncology [Internet]. 2020 [cited 2023 Oct 11];10. Available from: https://www.frontiersin.org/articles/10.3389/fonc.2020.00727

52. Zhang L, Smyth D, Al-Khalaf M, Blet A, Du Q, Bernick J, et al. Insulin-like growth factor-binding protein-7 (IGFBP7) links senescence to heart failure. Nat Cardiovasc Res. 2022;1:1195–214.

53. Houtman E, Almeida RC de, Tuerlings M, Suchiman HED, Broekhuis D, Nelissen RGHH, et al. Characterization of dynamic changes in Matrix Gla Protein (MGP) gene expression as function of genetic risk alleles, osteoarthritis relevant stimuli, and the vitamin K inhibitor warfarin. Osteoarthritis and Cartilage. 2021;29:1193–202.

54. Mertsch S, Schurgers LJ, Weber K, Paulus W, Senner V. Matrix gla protein (MGP): an overexpressed and migration-promoting mesenchymal component in glioblastoma. BMC Cancer. 2009;9:302.

55. Kim H-T, Yin W, Jin Y-J, Panza P, Gunawan F, Grohmann B, et al. Myh10 deficiency leads to defective extracellular matrix remodeling and pulmonary disease. Nat Commun. 2018;9:4600.

56. Kislev N, Mor-Yossef Moldovan L, Barak R, Egozi M, Benayahu D. MYH10 Governs Adipocyte Function and Adipogenesis through Its Interaction with GLUT4. Int J Mol Sci. 2022;23:2367.

57. Haraguchi R, Kitazawa R, Mori K, Tachibana R, Kiyonari H, Imai Y, et al. sFRP4-dependent Wnt signal modulation is critical for bone remodeling during postnatal development and age-related bone loss. Sci Rep. 2016;6:25198.

58. Pawar NM, Rao P. Secreted frizzled related protein 4 (sFRP4) update: A brief review. Cellular Signalling. 2018;45:63–70.

59. Kuo DS, Labelle-Dumais C, Gould DB. COL4A1 and COL4A2 mutations and disease: insights into pathogenic mechanisms and potential therapeutic targets. Human Molecular Genetics. 2012;21:R97–110.

60. Wang T, Jin H, Hu J, Li X, Ruan H, Xu H, et al. COL4A1 promotes the growth and metastasis of hepatocellular carcinoma cells by activating FAK-Src signaling. Journal of Experimental & Clinical Cancer Research. 2020;39:148.

61. Mondal M, Conole D, Nautiyal J, Tate EW. UCHL1 as a novel target in breast cancer: emerging insights from cell and chemical biology. Br J Cancer. 2022;126:24–33.

62. Nawaz MS, Asghar R, Pervaiz N, Ali S, Hussain I, Xing P, et al. Molecular evolutionary and structural analysis of human UCHL1 gene demonstrates the relevant role of intragenic epistasis in Parkinson’s disease and other neurological disorders. BMC Evolutionary Biology. 2020;20:130.

63. Milewicz DM, Østergaard JR, Ala-Kokko LM, Khan N, Grange DK, Mendoza-Londono R, et al. De novo ACTA2 mutation causes a novel syndrome of multisystemic smooth muscle dysfunction. American Journal of Medical Genetics Part A. 2010;152A:2437–43.

64. van de Laar IMBH, Arbustini E, Loeys B, Björck E, Murphy L, Groenink M, et al. European reference network for rare vascular diseases (VASCERN) consensus statement for the screening and management of patients with pathogenic ACTA2 variants. Orphanet Journal of Rare Diseases. 2019;14:264.

65. Gao Y, Xu Q, Li X, Guo Y, Zhang B, Jin Y, et al. Heterogeneity induced GZMA-F2R communication inefficient impairs antitumor immunotherapy of PD-1 mAb through JAK2/STAT1 signal suppression in hepatocellular carcinoma. Cell Death Dis. 2022;13:1–14.

66. Gigante B, Bellis A, Visconti R, Marino M, Morisco C, Trimarco V, et al. Retrospective Analysis of Coagulation Factor II Receptor (F2R) Sequence Variation and Coronary Heart Disease in Hypertensive Patients. Arteriosclerosis, Thrombosis, and Vascular Biology. 2007;27:1213–9.

67. Duan C, Allard JB. Insulin-Like Growth Factor Binding Protein-5 in Physiology and Disease. Frontiers in Endocrinology [Internet]. 2020 [cited 2023 Oct 11];11. Available from: https://www.frontiersin.org/articles/10.3389/fendo.2020.00100

68. Song C, Wang S, Fu Z, Chi K, Geng X, Liu C, et al. IGFBP5 promotes diabetic kidney disease progression by enhancing PFKFB3-mediated endothelial glycolysis. Cell Death Dis. 2022;13:1–12.

69. Osorio-Conles Ó, Guitart M, Moreno-Navarrete JM, Escoté X, Duran X, Fernandez-Real JM, et al. Adipose Tissue and Serum CCDC80 in Obesity and Its Association with Related Metabolic Disease. Mol Med. 2017;23:225–34.

70. Sasagawa S, Nishimura Y, Sawada H, Zhang E, Okabe S, Murakami S, et al. Comparative Transcriptome Analysis Identifies CCDC80 as a Novel Gene Associated with Pulmonary Arterial Hypertension. Frontiers in Pharmacology [Internet]. 2016 [cited 2023 Oct 11];7. Available from: https://www.frontiersin.org/articles/10.3389/fphar.2016.00142

71. An Q, Liu T, Wang M-Y, Yang Y-J, Zhang Z-D, Liu Z-J, et al. KRT7 promotes epithelial‑mesenchymal transition in ovarian cancer via the TGF‑β/Smad2/3 signaling pathway. Oncology Reports. 2021;45:481–92.

72. Polari L, Tenhami M, Anttila S, Helenius T, Kujari H, Kallajoki M, et al. Colonocyte keratin 7 is expressed de novo in inflammatory bowel diseases and associated with pathological changes and drug-resistance. Sci Rep. 2022;12:22213.

73. Wang C-S, Lin K-H, Chen S-L, Chan Y-F, Hsueh S. Overexpression of SPARC gene in human gastric carcinoma and its clinic–pathologic significance. Br J Cancer. 2004;91:1924–30.

74. Xu YZ, Heravi M, Thuraisingam T, Marco SD, Muanza T, Radzioch D. Brg-1 mediates the constitutive and fenretinide-induced expression of SPARC in mammary carcinoma cells via its interaction with transcription factor Sp1. Molecular Cancer. 2010;9:210.

75. Sah SK, Agrahari G, Kim T-Y. Insights into superoxide dismutase 3 in regulating biological and functional properties of mesenchymal stem cells. Cell & Bioscience. 2020;10:22.

76. Zachariae EDF, Hu L, Petersen SV. Chapter 12 - Extracellular superoxide dismutase (SOD3): An antioxidant or prooxidant in the extracellular space? In: Sies H, editor. Oxidative Stress [Internet]. Academic Press; 2020 [cited 2023 Oct 12]. p. 183–215. Available from: https://www.sciencedirect.com/science/article/pii/B9780128186060000122

77. Knight BE, Kozlowski N, Havelin J, King T, Crocker SJ, Young EE, et al. TIMP-1 Attenuates the Development of Inflammatory Pain Through MMP-Dependent and Receptor-Mediated Cell Signaling Mechanisms. Frontiers in Molecular Neuroscience [Internet]. 2019 [cited 2023 Oct 12];12. Available from: https://www.frontiersin.org/articles/10.3389/fnmol.2019.00220

78. Song G, Xu S, Zhang H, Wang Y, Xiao C, Jiang T, et al. TIMP1 is a prognostic marker for the progression and metastasis of colon cancer through FAK-PI3K/AKT and MAPK pathway. Journal of Experimental & Clinical Cancer Research. 2016;35:148.

79. Guvakova MA, Prabakaran I, Wu Z, Hoffman DI, Huang Y, Tchou J, et al. CDH2/N-cadherin and early diagnosis of invasion in patients with ductal carcinoma in situ. Breast Cancer Res Treat. 2020;183:333–46.

80. László ZI, Lele Z. Flying under the radar: CDH2 (N-cadherin), an important hub molecule in neurodevelopmental and neurodegenerative diseases. Frontiers in Neuroscience [Internet]. 2022 [cited 2023 Oct 12];16. Available from: https://www.frontiersin.org/articles/10.3389/fnins.2022.972059

81. Ershov PV, Mezentsev YV, Kopylov AT, Yablokov EO, Svirid AV, Lushchyk AYa, et al. Affinity Isolation and Mass Spectrometry Identification of Prostacyclin Synthase (PTGIS) Subinteractome. Biology (Basel). 2019;8:49.

82. Pan X, Yang Y, Meng H, Li H, Chen X, Huang H, et al. DNA Methylation of PTGIS Enhances Hepatic Stellate Cells Activation and Liver Fibrogenesis. Frontiers in Pharmacology [Internet]. 2018 [cited 2023 Oct 12];9. Available from: https://www.frontiersin.org/articles/10.3389/fphar.2018.00553

83. Li X, Zhao D, Guo Z, Li T, Qili M, Xu B, et al. Overexpression of SerpinE2/protease nexin-1 Contribute to Pathological Cardiac Fibrosis via increasing Collagen Deposition. Sci Rep. 2016;6:37635.

84. Yang Y, Xin X, Fu X, Xu D. Expression pattern of human SERPINE2 in a variety of human tumors. Oncology Letters. 2018;15:4523–30.

85. Xu X, Lu X, Chen L, Peng K, Ji F. Downregulation of MMP1 functions in preventing perineural invasion of pancreatic cancer through blocking the NT‐3/TrkC signaling pathway. J Clin Lab Anal. 2022;36:e24719.

86. Zhou J, Xu M, Tan J, Zhou L, Dong F, Huang T. MMP1 acts as a potential regulator of tumor progression and dedifferentiation in papillary thyroid cancer. Frontiers in Oncology [Internet]. 2022 [cited 2023 Oct 12];12. Available from: https://www.frontiersin.org/articles/10.3389/fonc.2022.1030590

87. Kim E-M, Hwang O. Role of matrix metalloproteinase-3 in neurodegeneration. Journal of Neurochemistry. 2011;116:22–32.

88. Wan J, Zhang G, Li X, Qiu X, Ouyang J, Dai J, et al. Matrix Metalloproteinase 3: A Promoting and Destabilizing Factor in the Pathogenesis of Disease and Cell Differentiation. Frontiers in Physiology [Internet]. 2021 [cited 2023 Oct 12];12. Available from: https://www.frontiersin.org/articles/10.3389/fphys.2021.663978

89. Alateyah N, Gupta I, Rusyniak RS, Ouhtit A. SOD2, a Potential Transcriptional Target Underpinning CD44-Promoted Breast Cancer Progression. Molecules. 2022;27:811.

90. Song J, Jiang L, Fu C, Wu X, Liu Z, Xie L, et al. Heterozygous SOD2 deletion deteriorated chronic intermittent hypoxia-induced lung inflammation and vascular remodeling through mtROS-NLRP3 signaling pathway. Acta Pharmacol Sin. 2020;41:1197–207.

91. Fei F, Qu J, Li C, Wang X, Li Y, Zhang S. Role of metastasis-induced protein S100A4 in human non-tumor pathophysiologies. Cell & Bioscience. 2017;7:64.

92. Helfman DM, Kim EJ, Lukanidin E, Grigorian M. The metastasis associated protein S100A4: role in tumour progression and metastasis. Br J Cancer. 2005;92:1955–8.

93. Liu Z, Gu S, Lu T, Wu K, Li L, Dong C, et al. IFI6 depletion inhibits esophageal squamous cell carcinoma progression through reactive oxygen species accumulation via mitochondrial dysfunction and endoplasmic reticulum stress. Journal of Experimental & Clinical Cancer Research. 2020;39:144.

94. Sajid M, Ullah H, Yan K, He M, Feng J, Shereen MA, et al. The Functional and Antiviral Activity of Interferon Alpha-Inducible IFI6 Against Hepatitis B Virus Replication and Gene Expression. Front Immunol. 2021;12:634937.

95. Geven C, Kox M, Pickkers P. Adrenomedullin and Adrenomedullin-Targeted Therapy As Treatment Strategies Relevant for Sepsis. Frontiers in Immunology [Internet]. 2018 [cited 2023 Oct 12];9. Available from: https://www.frontiersin.org/articles/10.3389/fimmu.2018.00292

96. Simonyte S, Kuciene R, Dulskiene V, Lesauskaite V. Associations of the adrenomedullin gene polymorphism with prehypertension and hypertension in Lithuanian children and adolescents: a cross-sectional study. Sci Rep. 2019;9:6807.

97. Vorkapic E, Kunath A, Wågsäter D. Effects of osteoprotegerin/TNFRSF11B in two models of abdominal aortic aneurysms. Molecular Medicine Reports. 2018;18:41–8.

98. Xu M, Zhang C, Han Y, Yue Z, Shu C, Hou J. Association between Osteoprotegerin rs2073618 polymorphism and peri-implantitis susceptibility: a meta-analysis. BMC Oral Health. 2022;22:598.

99. Gao L, Li S, Yue H, Zhang Z. Associations of Serum Cathepsin K and Polymorphisms in CTSK Gene With Bone Mineral Density and Bone Metabolism Markers in Postmenopausal Chinese Women. Frontiers in Endocrinology [Internet]. 2020 [cited 2023 Oct 12];11. Available from: https://www.frontiersin.org/articles/10.3389/fendo.2020.00048

100. Ho N, Punturieri A, Wilkin D, Szabo J, Johnson M, Whaley J, et al. Mutations of CTSK Result in Pycnodysostosis via a Reduction in Cathepsin K Protein. Journal of Bone and Mineral Research. 1999;14:1649–53.

101. Guyon A. CXCL12 chemokine and its receptors as major players in the interactions between immune and nervous systems. Frontiers in Cellular Neuroscience [Internet]. 2014 [cited 2023 Oct 12];8. Available from: https://www.frontiersin.org/articles/10.3389/fncel.2014.00065

102. Janssens R, Struyf S, Proost P. The unique structural and functional features of CXCL12. Cell Mol Immunol. 2018;15:299–311.

103. Karnaukhov V, Paes W, Woodhouse IB, Partridge T, Nicastri A, Brackenridge S, et al. HLA variants have different preferences to present proteins with specific molecular functions which are complemented in frequent haplotypes. Front Immunol. 2022;13:1067463.

104. Yang X, Garner LI, Zvyagin IV, Paley MA, Komech EA, Jude KM, et al. Autoimmunity-associated T cell receptors recognize HLA-B*27-bound peptides. Nature. 2022;612:771–7.

105. Dzimianski JV, Scholte FEM, Bergeron É, Pegan SD. ISG15: It’s Complicated. Journal of Molecular Biology. 2019;431:4203–16.

106. Perng Y-C, Lenschow DJ. ISG15 in antiviral immunity and beyond. Nat Rev Microbiol. 2018;16:423–39.

107. Cheng C-Y, Zhou Z, Stone M, Lu B, Flesken-Nikitin A, Nanus DM, et al. Membrane metalloendopeptidase suppresses prostate carcinogenesis by attenuating effects of gastrin-releasing peptide on stem/progenitor cells. Oncogenesis. 2020;9:1–14.

108. Prausmüller S, Spinka G, Arfsten H, Stasek S, Rettl R, Bartko PE, et al. Relevance of Neutrophil Neprilysin in Heart Failure. Cells. 2021;10:2922.

109. Almarán B, Ramis G, Fernández de Mattos S, Villalonga P. Rnd3 Is a Crucial Mediator of the Invasive Phenotype of Glioblastoma Cells Downstream of Receptor Tyrosine Kinase Signalling. Cells. 2022;11:3716.

110. Dankel SN, Røst TH, Kulyté A, Fandalyuk Z, Skurk T, Hauner H, et al. The Rho GTPase RND3 regulates adipocyte lipolysis. Metabolism - Clinical and Experimental [Internet]. 2019 [cited 2023 Oct 12];101. Available from: https://www.metabolismjournal.com/article/S0026-0495(19)30214-8/fulltext

111. Krishnaswamy VR, Korrapati PS. Role of Dermatopontin in re-epithelialization: Implications on keratinocyte migration and proliferation. Sci Rep. 2014;4:7385.

112. Xi L-C, Ji Y-X, Yin D, Zhao Z-X, Huang S-C, Yu S-L, et al. Effects of Dermatopontin gene silencing on apoptosis and proliferation of osteosarcoma MG‑63 cells. Molecular Medicine Reports. 2018;17:422–7.

113. Li X, Tao X, Ding X. An integrative analysis to reveal that CLEC2B and ferroptosis may bridge the gap between psoriatic arthritis and cancer development. Sci Rep. 2022;12:14653.

114. Zhang X, Yuan J, Wang X, Fu S, Wang R, Wang G. Association between c-type lectin-like receptor 2 and microsatellite instability in colorectal cancer: a cross-sectional study. BMC Cancer. 2022;22:823.

115. Goizet C, Boukhris A, Durr A, Beetz C, Truchetto J, Tesson C, et al. CYP7B1 mutations in pure and complex forms of hereditary spastic paraplegia type 5. Brain. 2009;132:1589–600.

116. Yantsevich AV, Dichenko YV, MacKenzie F, Mukha DV, Baranovsky AV, Gilep AA, et al. Human steroid and oxysterol 7α-hydroxylase CYP7B1: substrate specificity, azole binding and misfolding of clinically relevant mutants. The FEBS Journal. 2014;281:1700–13.

117. Franco HL, Casasnovas J, Rodríguez-Medina JR, Cadilla CL. Redundant or separate entities?—roles of Twist1 and Twist2 as molecular switches during gene transcription. Nucleic Acids Research. 2011;39:1177–86.

118. Gaulton N, Wakelin G, Young LV, Wotherspoon S, Kamal M, Parise G, et al. Twist2-expressing cells reside in human skeletal muscle and are responsive to aging and resistance exercise training. The FASEB Journal. 2022;36:e22642.

119. Gschwandtner M, Derler R, Midwood KS. More Than Just Attractive: How CCL2 Influences Myeloid Cell Behavior Beyond Chemotaxis. Frontiers in Immunology [Internet]. 2019 [cited 2023 Oct 11];10. Available from: https://www.frontiersin.org/articles/10.3389/fimmu.2019.02759

120. Hao Q, Vadgama JV, Wang P. CCL2/CCR2 signaling in cancer pathogenesis. Cell Communication and Signaling. 2020;18:82.

121. Pantazatos SP, Huang Y, Rosoklija GB, Dwork AJ, Arango V, Mann JJ. Whole-transcriptome brain expression and exon-usage profiling in major depression and suicide: evidence for altered glial, endothelial and ATPase activity. Mol Psychiatry. 2017;22:760–73.

122. Shen Y, Peng C, Bai Q, Ding Y, Yi X, Du H, et al. Epigenome-Wide Association Study Indicates Hypomethylation of MTRNR2L8 in Large-Artery Atherosclerosis Stroke. Stroke. 2019;50:1330–8.

123. Bourin P, Bunnell BA, Casteilla L, Dominici M, Katz AJ, March KL, et al. Stromal cells from the adipose tissue-derived stromal vascular fraction and culture expanded adipose tissue-derived stromal/stem cells: a joint statement of the International Federation for Adipose Therapeutics and Science (IFATS) and the International Society for Cellular Therapy (ISCT). Cytotherapy. 2013;15:641–8.

124. Dominici M, Le Blanc K, Mueller I, Slaper-Cortenbach I, Marini F, Krause D, et al. Minimal criteria for defining multipotent mesenchymal stromal cells. The International Society for Cellular Therapy position statement. Cytotherapy. 2006;8:315–7.

125. Krampera M, Galipeau J, Shi Y, Tarte K, Sensebe L, MSC Committee of the International Society for Cellular Therapy (ISCT). Immunological characterization of multipotent mesenchymal stromal cells--The International Society for Cellular Therapy (ISCT) working proposal. Cytotherapy. 2013;15:1054–61.

126. Viswanathan S, Shi Y, Galipeau J, Krampera M, Leblanc K, Martin I, et al. Mesenchymal stem versus stromal cells: International Society for Cell & Gene Therapy (ISCT®) Mesenchymal Stromal Cell committee position statement on nomenclature. Cytotherapy. 2019;21:1019–24.

127. Ascensión AM, Fuertes-Álvarez S, Ibañez-Solé O, Izeta A, Araúzo-Bravo MJ. Human Dermal Fibroblast Subpopulations Are Conserved across Single-Cell RNA Sequencing Studies. Journal of Investigative Dermatology. 2021;141:1735-1744.e35.

128. Lendahl U, Muhl L, Betsholtz C. Identification, discrimination and heterogeneity of fibroblasts. Nat Commun. 2022;13:3409.

129. Lupatov AYu, Vdovin AS, Vakhrushev IV, Poltavtseva RA, Yarygin KN. Comparative Analysis of the Expression of Surface Markers on Fibroblasts and Fibroblast-Like Cells Isolated from Different Human Tissues. Bull Exp Biol Med. 2015;158:537–43.

130. Plikus MV, Wang X, Sinha S, Forte E, Thompson SM, Herzog EL, et al. Fibroblasts: origins, definitions, and functions in health and disease. Cell. 2021;184:3852–72.
